# Copper/*N,N*-Dimethylglycine Catalyzed Goldberg Reactions Between Aryl Bromides and Amides, Aryl Iodides and Secondary Acyclic Amides

**DOI:** 10.3390/molecules190913448

**Published:** 2014-08-29

**Authors:** Liqin Jiang

**Affiliations:** Shanghai Engineering Research Center of Molecular Therapeutics and New Drug Development, East China Normal University, 3663 North Zhongshan Road, Shanghai 200062, China; E-Mail: jiangliqin_777@163.com or lqjiang@sat.ecnu.edu.cn; Tel.: +86-21-6223-2468; Fax: +86-21-5434-1159

**Keywords:** *N,N*-dimethylglycine, Goldberg reaction, aryl bromides, secondary acyclic amides

## Abstract

An efficient and general copper-catalyzed Goldberg reaction at 90–110 °C between aryl bromides and amides providing the desired products in good to excellent yields has been developed using *N,N*-dimethylglycine as the ligand. The reaction is tolerant toward a wide range of amides and a variety of functional group substituted aryl bromides. In addition, hindered, unreactive aromatic and aliphatic secondary acyclic amides, known to be poor nucleophiles, are efficiently coupled with aryl iodides through this simple and cheap copper/*N,N*-dimethylglycine catalytic system.

## 1. Introduction

*N*-arylation of amides has received considerable attention over the past decades due to its important synthetic utility [1,2,3,4]. Although Pd-catalyzed arylations of amides have made great progress [2,3,5,6,7,8,9,10] the high cost of Pd and the corresponding ligands as well as the difficulty of removing the Pd from polar products limit their application. The less expensive copper-catalyzed *N*-arylation of amides, known as the Goldberg reacton, is still an attractive option. Significant improvements have been achieved in the Goldberg reaction over the past decade by the introduction of chelating ligands, which make this transformation work under significantly milder reaction conditions [3]. For instance, in 2002, Buchwald and coworkers reported that several diamine-type ligands could greatly facilitate the coupling of aryl halides with amides at low temperatures [11,12]. This catalyst system was later extended to other coupling partners such as carbamates [13], iodo-selenophene [14], halo-furans [15], and oxindoles [16]. However, one of diamine ligands, trans-*N,N′*-dimethyl-1,2-cyclohexanediamine, is more expensive than other common ligands. Moreover, even for the coupling of aryl iodides and hindered, unreactive amide substrates with the diamine-type ligands, significant *N*-arylation of the diamine ligand (>5% with respect to the aryl halide) is observed. The resulting *N*-arylated diamines are less catalytically active than the starting diamine, a problem which is further exacerbated in the reactions involving hindered amides [12]. Most of the subsequently reported copper/ligand catalytic systems for the Goldberg reaction only can be applied to the coupling of aryl iodides and amides [14,17,18,19,20,21,22]. To the best of our knowledge, besides diamine ligands, only β-keto ester [23] and amino acid ligands [24,25] were applicable to the copper catalyzed coupling of aryl bromides and amides and the amide substrates were restricted to primary amides [24], lactams [23] and oxazolidinones [25]. A systematical study of the coupling of aryl bromides with diversified amides, especially with acyclic secondary amides and amino acids as ligands has not been reported yet. Tertiary amides which could be generated from aryl halides and secondary amides are found in numerous biologically active compounds [26,27,28,29,30]. Synthesis of tertiary aryl amides through arylation of the secondary amides would facilitate the generation of analogues for the structure-activity study in medicinal chemistry and life science by easily changing the aryl component. Secondary amides are often intermediates in total synthesis and their direct arylation would facilitate more efficient synthetic routes [30]. Additionally, provided that we sequentially arylated primary amides and *N*-alkylated the resulting *N*-aryl amides to prepare the tertiary aryl amides, dialkylation resulting in quaternary ammonium salts, or *N,O*-dialkylation could occur. Only a few reactions involving palladium or copper as catalysts for *N*-arylation of secondary acyclic amides have been described [10,22]. Cheap, general, efficient catalyst systems for *N*-arylation of secondary amides under mild conditions are still desirable. As a result of our interest in the ligand-promoted Ullman and Goldberg reactions [31], we found that CuI/*N,N*-dimethylglycine is a cheap, efficient and general catalytic system for the coupling of aryl bromides and amides, affording the corresponding products, including diversified tertiary amides, in good to excellent yields at 90–110 °C. In addition, one of the advantages of using *N,N*-dimethylglycine as a ligand is that it cannot be arylated. This simple and cheap catalytic system also gave good yields when hindered, unreactive amide substrates were coupled with aryl iodides. Herein, we report in details our results.

## 2. Results and Discussion

As indicated in Table 1, a CuI-catalyzed coupling reaction of 1-bromo-4-methoxybenzene (**1a**) with *N*-methylformamide (**2a**) was chosen as a model reaction to screen suitable ligands. Given that 4-hydroxy-l-proline is an effective ligand for the coupling of *N*-substituted *o*-bromobenzamides with primary amides [24] and effective in other C-N coupling reactions [31,32], we initially carried out the model reaction with 10 mol% of CuI, 20 mol% of 4-hydroxy-l-proline and 2 equivalents of K_2_CO_3_ in 1 mL of DMF at 110 °C. The desired product was isolated in 24% yield after 24 h (Table 1, entry 1). Increasing the amount of the ligand to 30 mol% led to a negligible change of the yield (Table 1, entry 2). Increasing the concentration of the reaction by decreasing the amount of DMF to 0.5 mL improved the yield to 61% (Table 1, entry 3). Then other bases, solvents, and copper salts were screened (Table 1, entry 4–7). Using K_3_PO_4_ instead of K_2_CO_3_ as a base gave almost the same results (Table 1, entry 4). Cs_2_CO_3_ as base led to no product (Table 1, entry 5). Dioxane or DMSO as solvent decreased the yield (Table 1, entry 6, 7). Other copper salts such as copper(I) oxide and copper(II) acetylacetone also led to a decrease in the yield (Table 1, entry 8, 9). Then other 2-carboxylic acid bidentate compounds were examined as ligands for this model reaction under the above optimized reaction conditons (Table 1, entry 10–16). Only a trace amount of the desired product was detected with glycine as ligand instead of 4-hydroxy-L-proline, although glycine was reported to be an efficient ligand in promoting amidation of aryl iodides (Table 1, entry 10) [18]. Proline as a ligand in the current reaction gave 76% yield after 24 h (Table 1, entry 11). When picolinic acid was tested as a ligand the reaction proceeded slowly and afforded an 86% yield of the coupling product after 36h when the starting material **1a** was completely consumed (Table 1, entry 12). To our delight, *N,N*-dimethylglycine was discovered to be a powerful ligand to accelerate the reaction and an almost quatitative yield was achieved after 12 h (Table 1, entry 13). Decreasing the amount of *N*-methylformamide (**2a**) from 2 to 1.2 equivalents still gave an almost quantitative yield after 20 h (Table 1, entry 14).

Bicine was inefficient to this reaction (Table 1, entry 15). Dimethylethanolamine was also an inefficient ligand to this reaction (Table 1, entry 16), demonstrating the importance of the carboxylic acid in *N,N*-dimethylglycine. Control reactions with only copper or *N,N*-dimethylglycine as catalyst both gave no desired product (Table 1, entry 17, 18). Thus, 10 mol% of CuI, 20 mol% of *N,N*-dimethylglycine and 2 equivilent of K_2_CO_3_ as base in DMF was identified as the optimized reaction condition.

With the optimized conditions in hand, we examined the scope of the Goldberg reaction between aryl bromides and amides. The results are summarized in Table 2. Generally, the reaction afforded the corresponding products in good to excellent yields under 90–110 °C. The reaction can be applicable to a wide range of amides, including primary amides (Table 2, entries 1–5), lactams (Table 2, entries 6–10) and secondary acyclic amides (Table 2, entries 11–18), as well as an *á,â*-unsaturated amide (Table 2, entry 5). Aryl bromides can contain strongly electron-donating *para*-substituents. For example, excellent yields were achieved when 1-bromo-4-methoxybenzene was amidated by either primary or secondary amides (enties 1, 5, 6, and 13). Free amine (entry 2) substitution on aryl bromides were tolerated, as well as acyl (entry 7, 15), fluoro (entry 12), trifluoromethyl (entries 8, 10). The bromo group in 4-bromo-1-chlorobenzene can be selectively amidated in the presence of the chloro substituent (entry 4). The method can also be extended to heteroaryl bromides such as 2-bromopyridine, 3-bromothiophene (entry 11, 17, 18). The latter is a substrate only moderately tolerant with the Pd-catalyzed methodology [33,34]. Heteroaromatic reagents are challenging because the heteroatom could ligate the catalyst, leading to catalyst deactivation, and also may be unfavorable for the catalytic processes [4]. When 1-bromo-2-methylbenzene was coupled with pyrrolidin-2-one the yield decreased to 76% (Table 2, entry 9). In order to solve this problem, we attempted to purify the CuI by another reported method [35]. We found that the yield can be improved to 91% using 10 mol% *N,N*-dimethylglycine and 10 mol% CuI purified by the dissolution-precipitation process [35]. Examining the model reaction of 1-bromo-4-methoxybenzene with *N*-methylformamide using 5% of CuI purified by this method and 10% *N,N*-dimethylglycine under 110 °C for 24h, a 95% yield was obtained. Other several reactions of aryl bromides with secondary acyclic amides were conducted under these conditions, and good to excellent yields were also achieved (entries 12,13,16,17).

**Table 1:** Optimization of Goldberg reaction conditions ^a^. 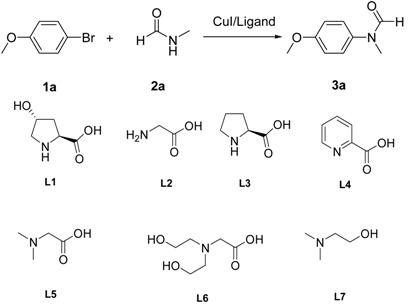

Entry	Copper Salt	Ligand	Solvent	Base	Yield ^b^ [%]
1	CuI	L1	DMF	K_2_CO_3_	24
2	CuI	L1 ^c^	DMF	K_2_CO_3_	25
3	CuI	L1	DMF ^d^	K_2_CO_3_	61
4	CuI	L1	DMF ^d^	K_3_PO_4_	60
5	CuI	L1	DMF ^d^	Cs_2_CO_3_	0
6	CuI	L1	DMSO ^d^	K_2_CO_3_	47
7	CuI	L1	Dioxane ^d^	K_2_CO_3_	42
8	Cu_2_O	L1	DMF ^d^	K_2_CO_3_	8
9	Cu(acac)_2_	L1	DMF ^d^	K_2_CO_3_	30
10	CuI	L2	DMF ^d^	K_2_CO_3_	trace
11	CuI	L3	DMF ^d^	K_2_CO_3_	76
12	CuI	L4	DMF ^d^	K_2_CO_3_	86 ^e^
13	CuI	L5	DMF ^d^	K_2_CO_3_	96 ^f^
14	CuI	L5	DMF ^d^	K_2_CO_3_	96 ^g^
15	CuI	L6	DMF ^d^	K_2_CO_3_	0
16	CuI	L7	DMF ^d^	K_2_CO_3_	0
17	CuI	--	DMF ^d^	K_2_CO_3_	0
18	--	L5	DMF ^d^	K_2_CO_3_	0

^a^ Reaction conditions: 10 mol% of CuI, 20 mol% of ligand, 1.0 equiv of 1-bromo-4-methoxybenzene, 2.0 equiv of *N*-methylformamide, 2 equiv of base in 1 mL of DMF, 24 h; ^b^ Isolated yield; ^c^ 30 mmol% of A; ^d^ Using 0.5 mL of solvent; ^e^ 36 h. ^f^ 12 h. ^g^ 1.2 equiv of *N*-methylformamide, 20 h.

Taillefer reported a copper-catalyzed coupling of aryl iodides with secondary acyclic amides under 130 °C using 2,2,6,6-tetramethyl-3,5-heptadione as ligand [22] as well as other procedures to afford tertiary amides from secondary amides and aryl iodides have been described [36]. Buchwald and coworkers report that diamine ligands are arylated when hindered, unreactive amide substrates are used, and the resulting *N*-arylated diamines are less catalytically active than the starting diamine, further aggravating the reactions involving hindered amides. The structure of *N,N*-dimethylglycine determined that it cannot be *N*-arylated in the coupling reaction. Thus we are interested in investigating its use in the coupling reaction of hindered, unreactive amide substrates with aryl iodides.

**Table 2:** CuI/*N,N*-dimethylglycine catalyzed amidation of aryl bromides ^a^. 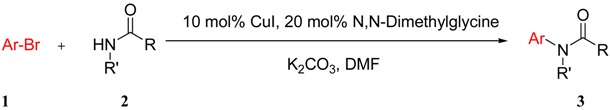

Entry		Product	3	T (°C)	t (h)		Yield ^b^ (%)	
1		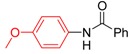	**3b**	100	22		96	
2		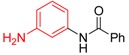	**3c**	100	24		92	
3		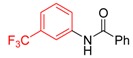	**3d**	100	16		93	
4		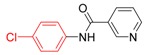	**3e**	100	8		97	
5		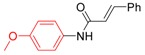	**3f**	100	20		90	
6		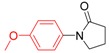	**3g**	90	18		90	
7		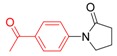	**3h**	90	16		93	
8		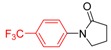	**3i**	100	16		83	
9		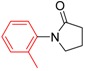	**3g**	110	24		76 91 ^f^	
10		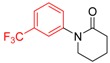	**3k**	110	26		91	
11		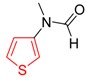	**3l**	110	24		95 ^c^	
12		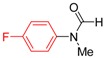	**3m**	100	19		93 93 ^f^	
13		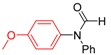	**3n**	110	20		95 93 ^f^	
14		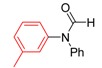	**3o**	90	19		94	
15	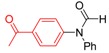		**3p**	110		27		90
16	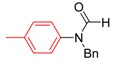		**3q**	110		14		96 92 ^f^
17	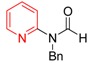		**3r**	100		18		71 ^d^ 70 ^f^
18	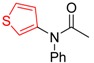		**3s**	110		24		74

^a^ Reaction conditions: 10 mol% of CuI, 20 mol% of *N,N*-dimethylglycine, 1.0 mmol of aryl bromide, 1.2 mmol of amide, 2 equiv of K_2_CO_3_ in 0.5 mL of DMF, 24 h; ^b^ Isolated yields; ^c^ With 2 equive of K_3_PO_4_ as base; ^d^ With 1.3 equive of aryl bromide and 1.0 equiv of amide; ^e^ With 2 equiv of amide substrates; ^f^ 5 mol% of dissolution-precipitation purified CuI, 10 mol% of *N,N*-dimethylglycine under 110 °C for 24 h.

**Table 3:** CuI/*N,N*-dimethylglycine catalyzed the coupling between hindered, unreactive amides and aryl iodides ^a^. 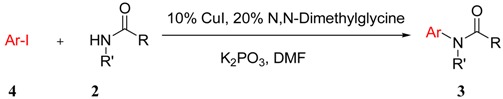

Entry	Product	3	T ( °C)	t (h)	Yield ^b^ (%)
1	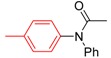	**3t**	90	24	85
2	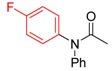	**3u**	80	24	81
3	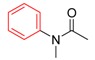	**3v**	90	24	82
4	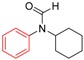	**3v**	90	24	82
5	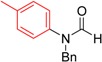	**3q**	80	24	92

^a^ Reaction conditions: 10 mol% of CuI, 20 mol% of *N,N*-dimethylglycine, 1.0 mmol of aryl iodides, 1.2 mmol of amide, 2 equiv of K_2_PO_3_ in 0.5 mL of DMF, 24 h. ^b^ Isolated yields.

As indicated in Table 3, *N*-phenylacetamide can be smoothly coupled with electron-donating or electron-drawing substituted aryl iodides at 80–90 °C under our catalyst system (Table 3, entry 1, 2). *N*-methylacetamide can also be arylated with iodobenzene at 90 °C, giving a good yield (Table 3, enty 3). The coupling reaction of bulky *N*-cyclohexylformamide with 1-iodobenzene afforded the corresponding product in 88% yield at 120 °C, which is higher than the reported 63% yield using diamine ligand [18] (Table 3, entry 4). When *N*-benzylformamide was coupled with 1-iodo-4-methylbenzene, an excellent yield was obtained at 80 °C (Table 3, entry 5).

## 3. Experimental Section

### 3.1. General Information & Materials

The new compounds were identified by ^1^H-NMR, ^13^C-NMR and HRMS. All ^1^H-NMR (300 MHz) and ^13^C-NMR (100 MHz) spectra were recorded on Bruker AMX-300, Varian EM360A spectrometers in CDCl_3_. Chemical shifts are reported in ppm relative to tetramethylsilane (δ = 0) or the residual protonated solvent (CDCl_3_: δ_H_ = 7.26 ppm, δ_C_ = 77.16 ppm). The resonance multiplicity is described as s (singlet), d (doublet), t (triplet), q (quartet), m (multiplet), br (broad). Mass spectra (EI-MS) were recorded on HP-5989A or VGQUATTRO mass spectrometers. High-resolution mass spectrometry (HRMS) was performed on a Bruker Daltonics FTMS-7 instrument. Melting points were uncorrected. DMF and DMSO were freshly distilled from CaH_2_. THF was distilled over sodium. Commercial available CuI should be washed with THF using a Soxhlet extractor before it was used to ensure the purity. When some reactions used the CuI purified according the reported method [35] comments and a note are provided in the paper. The reported compounds were identified by comparing with the reported analytical data by ^1^H-NMR and EI-MS.

### 3.2. General Procedure for Optimization of Amidation of Aryl Bromides with Copper/N,N-Dimethyl-glycine Catalytic System

A Schlenk tube was charged with 1-bromo-4-methoxybenzene **1a** (1 mmol), *N*-methylformamide **2a** (2 mmol or 1.2 mmol), CuI (0.1 mmol), ligand (0.2 mmol), and base (2 mmol). The tube was evacuated and backfilled with argon at room temperature. The solvent (0.5 mL) was added under argon via syringe. The Schlenk tube was immersed in a preheated oil bath and the reaction mixture was stirred at 110 °C for the specified time with complete consumption of the starting material **1a**. The cooled mixture was partitioned between water and ethyl acetate. The organic layer was separated and the aqueous layer was extracted with ethyl acetate. The combined organic layers were washed with brine, dried over Na_2_SO_4_ and concentrated *in vacuo*. The residue was purified by column chromatography on silica gel (eluting with 1:8 to 1:2 ethyl acetate/petroleum ether) to give the desired product.

### 3.3. General Procedure for the Coupling of Aryl Bromides with Amides Using Copper/N,N-Dimethyl-glycine Catalytic System

A Schlenk tube was charged with amide (1.2 mmol), aryl halide (1 mmol), CuI (0.05 or 0.1 mmol), *N,N*-dimethylglycine (0.1 or 0.2 mmol), and potassium carbonate (2 mmol). The tube was evacuated and backfilled with argon at room temperature. DMF (0.5 mL) was added under argon via syringe. The Schlenk tube was immersed in a preheated oil bath and the reaction mixture was stirred for the specified time at the indicated temperature. The cooled mixture was partitioned between water and ethyl acetate. The organic layer was separated and the aqueous layer was extracted with ethyl acetate. The combined organic layers were washed with brine, dried over Na_2_SO_4_, and concentrated *in vacuo*. The residue was purified by column chromatography on silica gel (eluting with 1:8 to 1:2 ethyl acetate/petroleum ether) to give the the desired *N*-aryl amides.

### 3.4. General Procedure for the Coupling of Aryl Iodides with Hindered Amides Using Copper/N,N-Dimethylglycine Catalytic System

A Schlenk tube was charged with amide (1.2 mmol), aryl iodides (1 mmol), CuI (0.1 mmol), *N,N*-dimethylglycine (0.2 mmol), and potassium phosphate (2 mmol). The tube was evacuated and backfilled with argon at room temperature. DMF (0.5 mL) was added under argon via syringe. The Schlenk tube was immersed in a preheated oil bath and the reaction mixture was stirred for the specified time at the indicated temperature. The cooled mixture was partitioned between water and ethyl acetate. The organic layer was separated and the aqueous layer was extracted with ethyl acetate. The combined organic layers were washed with brine, dried over Na_2_SO_4_, and concentrated *in vacuo*. The residue was purified by column chromatography on silica gel (eluting with 1:8 to 1:2 ethyl acetate/petroleum ether) to give the the desired *N*-aryl amides.

### 3.5. Analytical Data of Products

**Table:** 

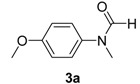	*N-(4-Methoxyphenyl)-N-methylformamide* (**3a**). ^1^H-NMR (CDCl_3_): δ 8.35 (s, 1H), 7.11 (d, *J* = 8.7 Hz, 2H), 6.93 (d, *J* = 9.0 Hz, 2H), 3.83 (s, 3H), 3.28 (s, 3H); EI-MS *m/z* 165 (M^+^), 153, 136, 122, 108, 94, 77, 65, 52.
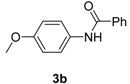	*N-(4-Methoxyphenyl)benzamide* (**3b**). ^1^H-NMR (CDCl_3_): δ 7.87 (d, *J* = 7.2 Hz, 1H ), 7.79 (s, 1H), 7.56–7.46 (m, 5H), 6.91 (d, *J* = 9.3 Hz, 2H), 3.82(s, 3H); EI-MS *m/z* 227 (M^+^), 210, 178, 149, 122, 105, 77, 51.
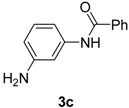	*N-(3-Aminophenyl)benzamide* (**3c**). ^1^H-NMR (CDCl_3_): δ 7.85 (d, *J* = 7.2 Hz, 2H), 7.79 (s, 1H), 7.57–7.45 (m, 3H), 7.33 (s, 1H), 7.13 ( t, *J* = 7.8 Hz, 1H), 6.79 (d, *J* = 7.8 Hz, 1H), 6.48 (d, *J_1_* = 8.1 Hz, *J_2_* = 1.8 Hz,1H), 3.71 (s, 2H); EI-MS *m/z* 212 (M^+^), 184, 167, 135, 121, 105, 77, 51.
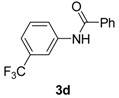	*N-(3-(Trifluoromethyl)phenyl)benzamide* (**3d**). ^1^H-NMR (CDCl_3_): δ 8.00–7.85 (m, 5H), 7.57–7.39 (m, 5H); EI-MS *m/z* 265 (M^+^), 246, 225, 149, 113, 105, 77, 51.
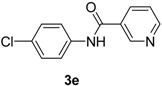	*N-(4-Chlorophenyl)nicotinamide* (**3e**). ^1^H-NMR (CDCl_3_): δ 9.08 (s, 1H), 8.78 (dd, *J_1_* = 4.8 Hz, *J_2_* = 1.5 Hz, 1H), 8.21 (d, *J* = 7.8 Hz, 1H), 8.04 (s, 1H), 7.62 (d, *J* = 9.0 Hz, 2H), 7.48–7.44 (m, 1H), 7.36 (d, *J* = 8.7 Hz, 2H); EI-MS *m/z* 232 (M^+^), 127, 106, 78, 51.
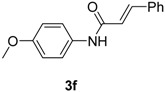	*N-(4-Methoxyphenyl)cinnamamide* (**3f**). ^1^H-NMR (CDCl_3_): δ 7.75 (d, *J* = 15.6 Hz, 1H), 7.54 (m, 4H), 7.38 (m, 3H), 6.90 (d, *J* = 8.7 Hz, 2H), 6.53 (d, *J* = 15.3 Hz, 1H), 3.81 (s, 3H); EI-MS *m/z* 253 (M^+^), 131, 123, 108, 103, 77, 51.
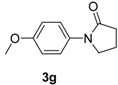	*1-(4-Methoxyphenyl)pyrrolidin-2-one* (**3g**). ^1^H-NMR (CDCl_3_): δ 7.50 (d, *J* = 9.0 Hz, 2H), 6.91 (d, *J* = 9.0 Hz, 2H), 3.86–3.00 (m, 5H), 2.60 (t, *J* = 8.1 Hz, 2H), 2.16 (m, 2H); EI-MS *m/z* 191 (M^+^), 176, 166, 136, 121, 69, 57.
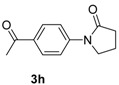	*1-(4-Acetylphenyl)pyrrolidin-2-one* (**3h**). ^1^H-NMR (CDCl_3_): δ 7.98 (d, *J* = 8.7 Hz, 2H), 7.76 (d, *J* = 9.0 Hz, 2H), 3.91 (t, *J* = 7.2 Hz, 2H), 2.65 (t, *J* = 7.5 Hz, 2H), 2.59 (s, 3H), 2.20 (m, 2H); EI-MS *m/z* 203 (M^+^), 188, 160, 148, 132, 120, 105, 90, 77, 69, 63, 51, 43.
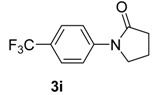	*1-(4-(Trifluoromethyl)phenyl)pyrrolidin-2-one* (**3i**). ^1^H-NMR (CDCl_3_): δ 7.77 (d, *J* = 8.4 Hz, 2H), 7.61 (d, *J* = 8.4 Hz, 2H), 3.89 (t, *J* = 7.2 Hz, 2H), 2.64 (t, *J* = 7.8 Hz, 2H), 2.21 (m, 2H); EI-MS *m/z* 229 (M^+^), 210, 174, 145, 127, 95, 84, 57.
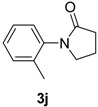	*1-o-Tolylpyrrolidin-2-one* (**3j**). ^1^H-NMR (CDCl_3_): δ 7.27–7.21 (m, 3H), 7.16–7.13 (m, 1H), 3.72 (t, *J* = 7.2 Hz, 2H), 2.59 (t, *J* = 8.1 Hz, 2H), 2.28–2.18 (m, 5H), EI-MS *m/z* 175 (M^+^), 158, 146, 130, 120, 91, 65, 51.
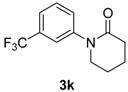	*1-(3-(Trifluoromethyl)phenyl)piperidin-2-one* (**3k**). ^1^H-NMR (CDCl_3_): δ 7.53–7.48 (m, 4H), 3.69–3.66 (m, 2H), 2.60–2.56 (m, 2H), 1.98–1.96 (m, 4H); EI-MS *m/z* 243 (M^+^), 224, 214, 187, 174, 149, 145, 120, 108, 91, 70, 55.
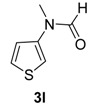	*N-Methyl-N-(thiophen-3-yl)formamide* (**3l**). ^1^H-NMR (CDCl_3_): δ 8.52 (s, 1H), 8.23 (s, 0.2H), 7.44–7.42 (m, 0.2H), 7.35–7.30 (m, 1.2 H), 7.25–7.23 (m, 0.2H), 6.99–6.97 (m, 1H), 6.84–6.83 (m, 1H), 3.30 (s, 0.6 H), 3.22 (s, 3H); EI-MS *m/z* 141 (M^+^), 112, 98, 85, 80, 72, 68, 58, 54, 45, 42.
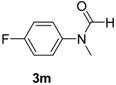	*N-(4-Fluorophenyl)-N-methylformamide* (**3m**). ^1^H-NMR (CDCl_3_): δ 8.40 (s, 1H), 7.18–7.12 (m, 4H), 3.31 (s, 3H); EI-MS *m/z* 153 (M^+^), 124, 112, 95, 83, 77, 75, 57.
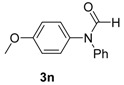	*N-(4-Methoxyphenyl)-N-phenylformamide* **(3n**). ^1^H-NMR (CDCl_3_): δ 8.62 (d, *J* = 30.9 Hz, 1H), 7.38–7.34 (m, 2H), 7.31–7.19 (m, 3H), 7.16–7.11 (m, 2H), 6.92 (d, *J* = 9.0 Hz, 2H), 3.81 (d, *J* = 4.2 Hz, 3H); EI-MS *m/z* 227 (M^+^), 199, 184, 167, 154, 129, 124, 121, 103, 93, 77, 66, 51, 43
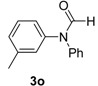	*N-Phenyl-N-m-tolylformamide* (**3o**). ^1^H-NMR (CDCl_3_): δ 8.66 (d, *J* = 6.3 Hz,1H), 7.43–7.38 (m, 2H), 7.33–7.26 (m, 3H), 7.18–7.05 (m, 3H), 6.98 (s, 1H), 2.36–2.35 (d, *J* = 3.6 Hz, 3H); EI-MS *m/z* 211 (M^+^), 183, 167, 141, 128, 118, 108, 91, 80, 65, 51; EI-HRMS for C_15_H_13_NO_2_ (M^+^) requires 211.0997, found 211.0992.
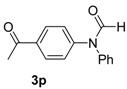	*N-(4-Acetylphenyl)-N-phenylformamide* (**3p**). ^1^H-NMR (CDCl_3_): δ 7.86 (d, *J* = 8.1 Hz, 2H), 7.34 (t, *J* = 7.5 Hz, 2H), 7.18 (d, *J* = 7.5 Hz, 2H), 7.08 (t, *J* = 7.2 Hz, 1H), 6.99 (d, *J* = 8.4 Hz, 2H), 6.26 (s, 1H), 2.63 (s, 3H); ^13^C-NMR (CDCl_3_): δ 196.3, 148.2, 140.4, 130.5 (2C), 129.4 (2C), 128.9, 123.2, 120.5 (2C), 114.2 (2C), 97.6, 26.0; EI-MS *m/z* 239 (M^+^), 211, 196, 167, 139, 115, 98, 84, 77, 63, 51, 43; EI-HRMS for C_15_H_13_NO_2_ (M^+^) requires 239.0946, found 239.0944.
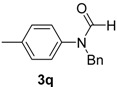	*N-Benzyl-N-p-tolylformamide* (**3q**). ^1^H-NMR (CDCl_3_): δ 8.50 (s, 1H), 7.28–7.21 (m, 5H), 7.13 (d, *J* = 8.1 Hz, 2H), 6.98 (d, *J* = 8.4 Hz, 2H), 4.97 (s, 2H), 2.32 (s, 3H); EI-MS *m/z* 225 (M^+^), 196, 181, 165, 133, 118, 91, 77, 65, 51
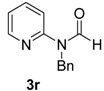	*N-Benzyl-N-(pyridin-2-yl)formamide* (**3r**). ^1^H-NMR (CDCl_3_): δ 9.50 (s, 1H), 8.39 (d, *J* = 5.1 Hz, 1H), 7.62 (t, *J* = 7.2 Hz, 1H), 7.29–7.22 (m, 5H), 7.08 (m, 1H), 6.93(d, *J* = 8.1 Hz, 1H), 5.18 (s, 2H); EI-MS *m/z* 212 (M^+^), 183, 168, 154, 106, 91, 78, 65, 51
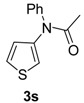	*N-Phenyl-N-(thiophen-3-yl)acetamide* (**3s**). ^1^H-NMR (CDCl_3_): δ 7.46–7.18 (m, 7H), 7.18 (6.95, *J* = 5.7 Hz, 1H), 1.99 (s, 3H); EI-MS *m/z* 217 (M^+^), 175, 149, 130, 120, 104, 84, 77, 51, 43.
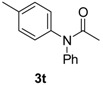	*N-(4-Fluorophenyl)-N-phenylacetamide* (**3t**). ^1^H-NMR (CDCl_3_): δ 7.32~7.15 (m, 9H), 2.33 (s, 3H), 2.05 (s, 3H).
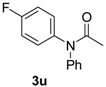	*N-(4-Fluorophenyl)-N-phenylacetamide* (**3u**). ^1^H-NMR (CDCl_3_): δ 7.40~6.99 (m, 9H), 2.06 (s, 3H).
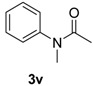	*N-methyl-N-phenylacetamide* (**3v**). ^1^H-NMR (CDCl_3_): δ 7.43 (t, *J* = 7.2 Hz, 2H), 7.34 (t, *J* = 7.2 Hz, 1H), 7.19 (d, *J* = 4.8 Hz, 2H), 3.27 (s, 3H), 1.87 (s, 3H); EI-MS *m/z* 149 (M^+^), 129, 120, 109, 106, 92, 81, 77, 65, 51, 43.
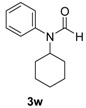	*N-Cyclohexyl-N-phenylformamide* (**3w**). ^1^H-NMR (CDCl_3_): δ 8.42 (s, 0.15H), 8.15 (s, 0.85H), 7.44–7.35 (m, 3H), 7.17–7.13 (m, 2H), 4.44–4.37 (m, 0.85H), 3.65–3.57 (m, 0.15H), 1.92–1.74 (m, 4H), 1.63–1.58 (m, 1H), 1.46–1.23 (m,4H), 1.05–0.95 (m, 1H); EI-MS *m/z* 203 (M^+^), 174, 160, 146, 132, 121, 118, 104, 93, 77, 66, 55, 51, 41.

## 4. Conclusions

In conclusion, we have found that CuI/*N,N*-dimethylglycine is a cheap, efficient and general catalytic system for the coupling of aryl bromides and amides, allowing for secondary acyclic amides to serve as substrates, and affording the corresponding products in good to excellent yields at 90–110 °C. A variety of functional groups are tolerated under the reaction conditions. In addition, It was noteworthy that unreactive, hindered aromatic and aliphatic secondary acyclic amides can also be efficiently coupled with aryl iodides under the CuI/*N,N*-dimethylglycine catalyst system. Based on the advantage of *N,N*-dimethylglycine which cannot be *N*-arylated or *N*-alkylated in the coupling reaction, the utility of it as a ligand to study other challenging coupling reactions are underway in our laboratory. (For NMR spectra of Products please see supplementary file).

## Supplementary Materials

Supplementary materials can be accessed at: http://www.mdpi.com/1420-3049/19/9/13448/s1.

## Supplementary Files

Supplementary File 1
