# Supplementary material for: Copper/N,N-Dimethylglycine Catalyzed Goldberg Reactions Between Aryl Bromides and Amides, Aryl Iodides and Secondary Acyclic Amides
Source: Molecules. 2014 Aug 29;19(9):13448–60. doi: 10.3390/molecules190913448 (PMC6271110; doi:10.3390/molecules190913448)
Supplement: Supplementary File 1 [file molecules-19-13448-s001.pdf]

Supplementary Materials

Contents

Supporting information S1

1. General Information & Materials S2

2. General Procedure for Optimization of Amidation of Aryl Bromides with Copper/*N,N*-Dimethylglycine Catalytic System S2

3. General Procedure for the Amidation of Aryl Bromides with Copper/*N,N*-Dimethylglycine Catalytic System. S2

4. General Procedure for the Amidation Of Aryl Iodides with Copper/N,N-Dimethylglycine Catalytic System. S3

5. Analytical Data of Products S3

6. Reference S5

7. NMR spectra of products S6

1. General Information & Materials

All ^{1}H NMR (300 MHz) and ^{13}C NMR (100 MHz) spectra were recorded on Brucker AMX-300, Varian EM360A spectrometers in $CDCl_3$. Tetramethylsilane (TMS) served as an internal standard ($\delta = 0$) for ^{1}H NMR, and $CDCl_3$ was used as internal standard ($\delta = 77.0$) for ^{13}C NMR. Chemical shifts are reported in parts per million as follows: chemical shift, multiplicity (s =singlet, d = doublet, t = triplet, q = quartet, m = multiplet, br = broad). Mass spectra (EI-MS) was performed on HP-5989A and VGQUATTRO mass spetrometry. High-resolution mass spectrometry (HRMS) was performed on Bruker Daltonics FTMS-7. Melting points were uncorrected. DMF and DMSO were freshly distilled from CaH_2. THF was distilled over sodium. Commercial available CuI should be washed with THF using a Soxhlet extractor before it is used. When some reactions used the CuI purified according the reported method [1], comments and note had been afforded in the paper. The reported compounds was identified by comparing with the reported analytical data by ^{1}H NMR (300 MHz) and EI-MS. The new compounds were identified by ^{1}H NMR (300 MHz), ^{13}C NMR (100 MHz) spectra, and HRMS.

2. General Procedure for Optimization of Amidation of Aryl Bromides with Copper/*N,N*-Dimethylglycine Catalytic System

A Schlenk tube was charged with 1-bromo-4-methoxybenzene **1a** (1 mmol), *N*-methylformamide **2a** (2 mmol or 1.2 mmol), CuI (0.1 mmol), ligand (0.2 mmol), and base (2 mmol). The tube was evacuated and backfilled with argon at room temperature. The solvent (0.5 mL) was added under argon via syringe. The Schlenk tube was immersed in a preheated oil bath and the reaction mixture was stirred at 110 °C for the specified time with complete consumption of the starting material **1a**. The cooled mixture was partitioned between water and ethyl acetate. The organic layer was separated and the aqueous layer was extracted with ethyl acetate. The combined organic layers were washed with brine, dried over Na_2SO_4, and concentrated in vacuo. The residual oil was loaded on a silica gel column and eluted with 1:8 to 1:2 ethyl acetate/petroleum ether to afford the corresponding *N*-aryl amide *N*-(4-methoxyphenyl)-*N*-methylformamide.

3. General Procedure for the Amidation of Aryl Bromides with Copper/*N,N*-Dimethylglycine Catalytic System

A Schlenk tube was charged with amide (1.2 mmol), aryl halide (1 mmol), CuI (0.05 or 0.1 mmol), *N,N*-dimethylglycine (0.1 or 0.2 mmol), and potassium carbonate (2 mmol). The tube was evacuated and backfilled with argon at room temperature. DMF (0.5 mL) was added under argon via syringe. The Schlenk tube was immersed in a preheated oil bath and the reaction mixture was stirred for the specified time at the indicated temperature. The cooled mixture was partitioned between water and ethyl acetate. The organic layer was separated and the aqueous layer was extracted with ethyl acetate. The combined organic layers were washed with brine, dried over Na_2SO_4, and concentrated in vacuum. The residual oil was loaded on a silica gel column and eluted with 1:8 to 1:2 ethyl acetate/ petroleum ether to afford the corresponding *N*-aryl amides.

4. General Procedure for the Amidation Of Aryl Iodides with Copper/*N,N*-Dimethylglycine Catalytic System

A Schlenk tube was charged with amide (1.2 mmol), aryl iodides (1 mmol), CuI (0.1 mmol), *N,N*-dimethylglycine (0.2 mmol), and potassium phosphate (2 mmol). The tube was evacuated and backfilled with argon at room temperature. DMF (0.5 mL) was added under argon via syringe. The Schlenk tube was immersed in a preheated oil bath and the reaction mixture was stirred for the specified time at the indicated temperature. The cooled mixture was partitioned between water and ethyl acetate. The organic layer was separated and the aqueous layer was extracted with ethyl acetate. The combined organic layers were washed with brine, dried over Na_2SO_4, and concentrated in vacuum. The residual oil was loaded on a silica gel column and eluted with 1:8 to 1:2 ethyl acetate/petroleum ether to afford the corresponding *N*-aryl amides.

5. Analytical Data of Products

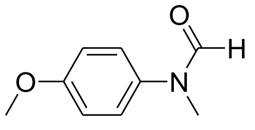

3a

N-(4-methoxyphenyl)-N-methylformamide (**3a**). ^{1}H NMR (300 MHz, $CDCl_3$): δ 8.35 (s, 1H), 7.11 (d, J = 8.7 Hz, 2H), 6.93 (d, J = 9.0 Hz, 2H), 3.83 (s, 3H), 3.28 (s, 3H); EI-MS *m/z* 165 (M^+), 153, 136, 122, 108, 94, 77, 65, 52.

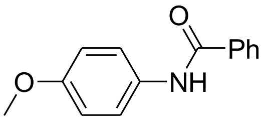

3b

N-(4-methoxyphenyl)benzamide (**3b**). ^{1}H NMR (300 MHz, $CDCl_3$): δ 7.87 (d, J = 7.2 Hz, 1H), 7.79 (s, 1H), 7.56-7.46 (m, 5H), 6.91 (d, J = 9.3 Hz, 2H), (3.82s, 3H); EI-MS *m/z* 227 (M^+), 210, 178, 149, 122, 105, 77, 51.

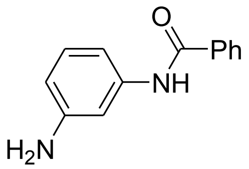

3c

N-(3-aminophenyl)benzamide (**3c**). ^{1}H NMR (300 MHz, $CDCl_3$): δ 7.85 (d, J = 7.2 Hz, 2H), 7.79 (s, 1H), 7.57-7.45 (m, 3H), 7.33 (s, 1H), 7.13 (t, J = 7.8 Hz, 1H), 6.79 (d, J = 7.8 Hz, 1H), 6.48 (d, J_1 = 8.1 Hz, J_2 = 1.8 Hz,1H), 3.71 (s, 2H); EI-MS *m/z* 212 (M^+), 184, 167, 135, 121, 105, 77, 51.

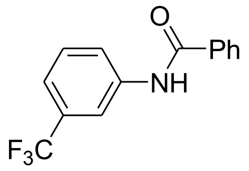

3d

N-(3-(trifluoromethyl)phenyl)benzamide (**3d**). ^{1}H NMR (300 MHz, $CDCl_3$): δ 8.00-7.85 (m, 5H), 7.57-7.39 (m, 5H); EI-MS *m/z* 265 (M^+), 246, 225, 149, 113, 105, 77, 51.

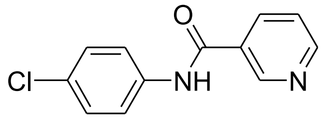

3e

N-(4-chlorophenyl)nicotinamide (**3e**). ^{1}H NMR (300 MHz, $CDCl_3$): δ 9.08 (s, 1H), 8.78 (dd, J_1 = 4.8 Hz, J_2 = 1.5 Hz, 1H), 8.21 (d, J = 7.8 Hz, 1H), 8.04 (s, 1H), 7.62 (d, J = 9.0 Hz, 2H), 7.48-7.44 (m, 1H), 7.36 (d, J = 8.7 Hz, 2H); EI-MS *m/z* 232 (M^+), 127, 106, 78, 51.

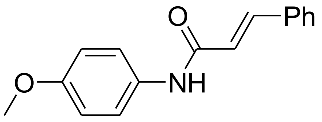

3f

N-(4-methoxyphenyl)cinnamamide (**3f**). ^{1}H NMR (300 MHz, $CDCl_3$): δ 7.75 (d, J = 15.6 Hz, 1H), 7.54 (m, 4H), 7.38 (m, 3H), 6.90 (d, J = 8.7 Hz, 2H), 6.53 (d, J = 15.3 Hz, 1H), 3.81 (s, 3H); EI-MS *m/z* 253 (M^+), 131, 123, 108, 103, 77, 51.

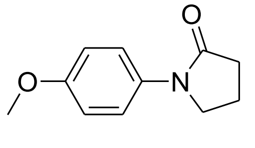 **3g**	*1-(4-methoxyphenyl)pyrrolidin-2-one* (**3g**). ^{1}H NMR (300 MHz, $CDCl_3$): δ 7.50 (d, *J* = 9.0 Hz, 2H), 6.91 (d, *J* = 9.0 Hz, 2H), 3.86-3.00 (m, 5H), 2.60 (t, *J* = 8.1 Hz, 2H), 2.16 (m, 2H); EI-MS *m/z* 191 (M^+), 176, 166, 136, 121, 69, 57.
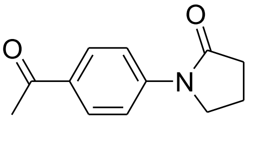 **3h**	*1-(4-acetylphenyl)pyrrolidin-2-one* (**3h**). ^{1}H NMR (300 MHz, $CDCl_3$): δ 7.98 (d, *J* = 8.7 Hz, 2H), 7.76 (d, *J* = 9.0 Hz, 2H), 3.91 (t, *J* = 7.2 Hz, 2H), 2.65 (t, *J* = 7.5 Hz, 2H), 2.59 (s, 3H), 2.20 (m, 2H); EI-MS *m/z* 203 (M^+), 188, 160, 148, 132, 120, 105, 90, 77, 69, 63, 51, 43.
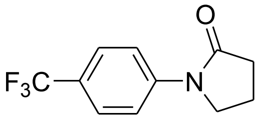 **3i**	*1-(4-(trifluoromethyl)phenyl)pyrrolidin-2-one* (**3i**). ^{1}H NMR (300 MHz, $CDCl_3$): δ 7.77 (d, *J* = 8.4 Hz, 2H), 7.61 (d, *J* = 8.4 Hz, 2H), 3.89 (t, *J* = 7.2 Hz, 2H), 2.64 (t, *J* = 7.8 Hz, 2H), 2.21 (m, 2H); EI-MS *m/z* 229 (M^+), 210, 174, 145, 127, 95, 84, 57.
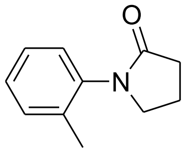 **3j**	*1-o-tolylpyrrolidin-2-one* (**3j**). ^{1}H NMR (300 MHz, $CDCl_3$): δ 7.27-7.21 (m, 3H), 7.16-7.13 (m, 1H), 3.72 (t, *J* = 7.2 Hz, 2H), 2.59 (t, *J* = 8.1 Hz, 2H), 2.28-2.18 (m, 5H), EI-MS *m/z* 175 (M^+), 158, 146, 130, 120, 91, 65, 51.
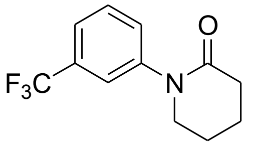 **3k**	*1-(3-(trifluoromethyl)phenyl)piperidin-2-one* (**3k**). ^{1}H NMR (300 MHz, $CDCl_3$): δ 7.53-7.48 (m, 4H), 3.69-3.66 (m, 2H), 2.60-2.56 (m, 2H), 1.98-1.96 (m, 4H); EI-MS *m/z* 243 (M^+), 224, 214, 187, 174, 149, 145, 120, 108, 91, 70, 55.
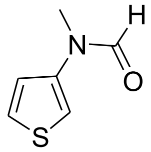 **3l**	*N-methyl-N-(thiophen-3-yl)formamide* (**3l**). ^{1}H NMR (300 MHz, $CDCl_3$): δ 8.52 (s, 1H), 8.23 (s, 0.2H), 7.44-7.42 (m, 0.2H), 7.35-7.30 (m, 1.2 H), 7.25-7.23 (m, 0.2H), 6.99-6.97 (m, 1H), 6.84-6.83 (m, 1H), 3.30 (s, 0.6 H), 3.22 (s, 3H); EI-MS *m/z* 141 (M^+), 112, 98, 85, 80, 72, 68, 58, 54, 45, 42.
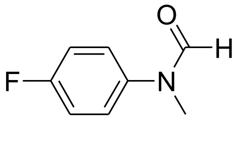 **3m**	*N-(4-fluorophenyl)-N-methylformamide* (**3m**). ^{1}H NMR (300 MHz, $CDCl_3$): δ 8.40 (s, 1H), 7.18-7.12 (m, 4H), 3.31 (s, 3H); EI-MS *m/z* 153 (M^+), 124, 112, 95, 83, 77, 75, 57.
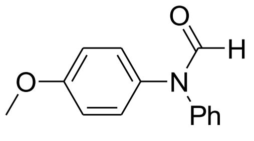 **3n**	*N-(4-methoxyphenyl)-N-phenylformamide* (**3n**). ^{1}H NMR (300 MHz, $CDCl_3$): δ 8.62 (d, *J* = 30.9 Hz, 1H), 7.38-7.34 (m, 2H), 7.31-7.19 (m, 3H), 7.16-7.11 (m, 2H), 6.92 (d, *J* = 9.0 Hz, 2H), 3.81 (d, *J* = 4.2 Hz, 3H); EI-MS *m/z* 227 (M^+), 199, 184, 167, 154, 129, 124, 121, 103, 93, 77, 66, 51, 43
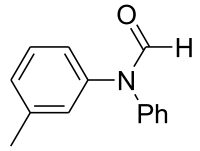 **3o**	*N-phenyl-N-m-tolylformamide* (**3o**). ^{1}H NMR (300 MHz, $CDCl_3$): δ 8.66 (d, *J* = 6.3 Hz,1H), 7.43-7.38 (m, 2H), 7.33-7.26 (m, 3H), 7.18-7.05 (m, 3H), 6.98 (s, 1H), 2.36-2.35 (d, *J* = 3.6 Hz, 3H); EI-MS *m/z* 211 (M^+), 183, 167, 141, 128, 118, 108, 91, 80, 65, 51; EI-HRMS for $C_{15}H_{13}NO_2$ (M^+) requires 211.0997, found 211.0992.

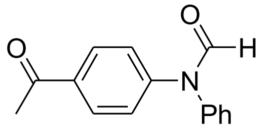 **3p**	*N-(4-acetylphenyl)-N-phenylformamide*. ^{1}H NMR (300 MHz, $CDCl_3$): δ 7.86 (d, *J* = 8.1 Hz, 2H), 7.34 (t, *J* = 7.5 Hz, 2H), 7.18 (d, *J* = 7.5 Hz, 2H), 7.08 (t, *J* = 7.2 Hz, 1H), 6.99 (d, *J* = 8.4 Hz, 2H), 6.26 (s, 1H), 2.63 (s, 3H); ^{13}C NMR (100 MHz, $CDCl_3$): δ 196.3, 148.2, 140.4, 130.5 (2C), 129.4 (2C), 128.9, 123.2, 120.5 (2C), 114.2 (2C), 97.6, 26.0; EI-MS *m/z* 239 (M^+), 211, 196, 167, 139, 115, 98, 84, 77, 63, 51, 43; EI-HRMS for $C_{15}H_{13}NO_2$ (M^+) requires 239.0946, found 239.0944.
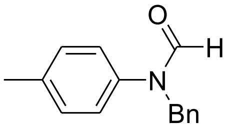 **3q**	*N-benzyl-N-p-tolylformamide* (**3q**). ^{1}H NMR (300 MHz, $CDCl_3$): δ 8.50 (s, 1H), 7.28-7.21 (m, 5H), 7.13 (d, *J* = 8.1 Hz, 2H), 6.98 (d, *J* = 8.4 Hz, 2H), 4.97 (s, 2H), 2.32 (s, 3H); EI-MS *m/z* 225 (M^+), 196, 181, 165, 133, 118, 91, 77, 65, 51
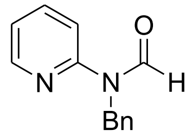 **3r**	*N-benzyl-N-(pyridin-2-yl)formamide* (**3r**). ^{1}H NMR (300 MHz, $CDCl_3$): δ 9.50 (s, 1H), 8.39 (d, *J* = 5.1 Hz, 1H), 7.62 (t, *J* = 7.2 Hz, 1H), 7.29-7.22 (m, 5H), 7.08 (m, 1H), 6.93(d, *J* = 8.1 Hz, 1H), 5.18 (s, 2H); EI-MS *m/z* 212 (M^+), 183, 168, 154, 106, 91, 78, 65, 51
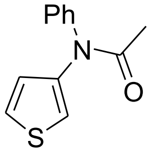 **3s**	*N-phenyl-N-(thiophen-3-yl)acetamide* (**3s**). ^{1}H NMR (300 MHz, $CDCl_3$): δ 7.46-7.18 (m, 7H), 7.18 (6.95, *J* = 5.7 Hz, 1H), 1.99 (s, 3H); EI-MS *m/z* 217 (M^+), 175, 149, 130, 120, 104, 84, 77, 51, 43.
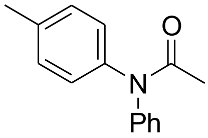 **3t**	*N-(4-fluorophenyl)-N-phenylacetamide* (**3t**). ^{1}H NMR (300 MHz, $CDCl_3$): δ 7.32~7.15 (m, 9H), 2.33 (s, 3H), 2.05 (s, 3H).
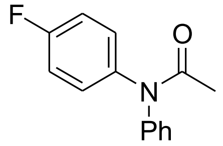 **3u**	*N-(4-fluorophenyl)-N-phenylacetamide* (**3u**). ^{1}H NMR (300 MHz, $CDCl_3$): δ 7.40~6.99 (m, 9H), 2.06 (s, 3H).
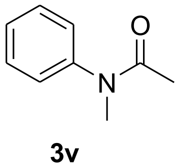 **3v**	*N-methyl-N-phenylacetamide* (**3v**). ^{1}H NMR (300 MHz, $CDCl_3$): δ 7.43 (t, *J* = 7.2 Hz, 2H), 7.34 (t, *J* = 7.2 Hz, 1H), 7.19 (d, *J* = 4.8 Hz, 2H), 3.27 (s, 3H), 1.87 (s, 3H); EI-MS *m/z* 149 (M^+), 129, 120, 109, 106, 92, 81, 77, 65, 51, 43.
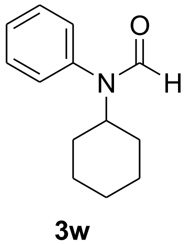 **3w**	*N-cyclohexyl-N-phenylformamide* (**3w**). ^{1}H NMR (300 MHz, $CDCl_3$): δ 8.42 (s, 0.15H), 8.15 (s, 0.85H), 7.44-7.35 (m, 3H), 7.17-7.13 (m, 2H), 4.44-4.37 (m, 0.85H), 3.65-3.57 (m, 0.15H), 1.92-1.74 (m, 4H), 1.63-1.58 (m, 1H), 1.46-1.23 (m,4H), 1.05-0.95 (m, 1H); EI-MS *m/z* 203 (M^+), 174, 160, 146, 132, 121, 118, 104, 93, 77, 66, 55, 51, 41.

6. Reference

1. Kauffman, G.B.; Fang, L.Y. *Inorganic Syntheses*; Holt, S.L., Ed.; Wiley: Hoboken, NJ, USA, 1983; Volume 22, pp. 101–103.

7. NMR Spectra of Products

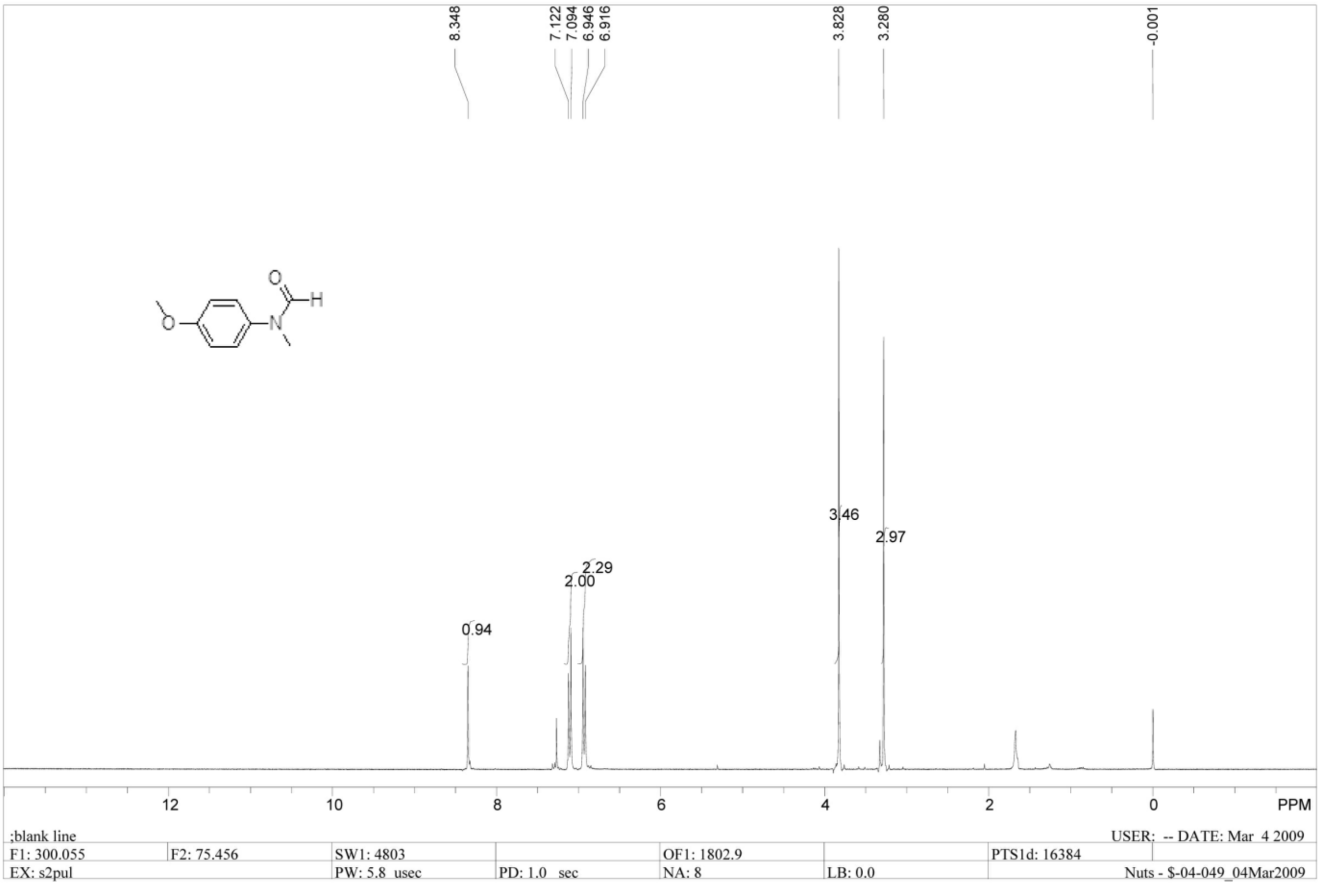

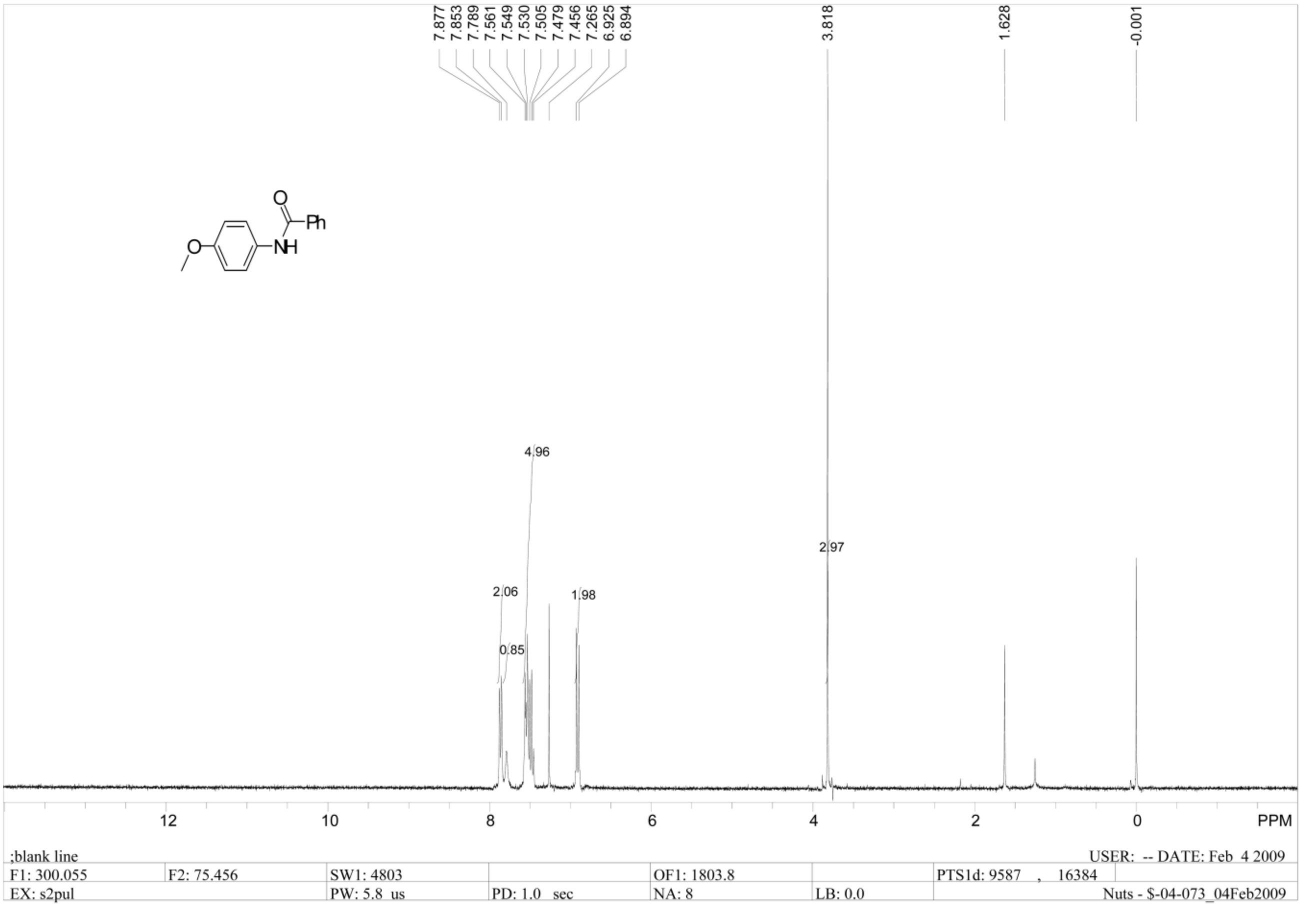
7.877
7.853
7.789
7.561
7.549
7.530
7.505
7.479
7.456
7.265
6.925
6.894
3.818
1.628
-0.001
O
Ph
NH
O
4.96
2.97
2.06
1.98
0.85
12
10
8
6
4
2
0
PPM
;blank line
USER: -- DATE: Feb 4 2009
F1: 300.055
F2: 75.456
SW1: 4803
OF1: 1803.8
PTS1d: 9587 , 16384
EX: s2pul
PW: 5.8 us
PD: 1.0 sec
NA: 8
LB: 0.0
Nuts - $-04-073_04Feb2009

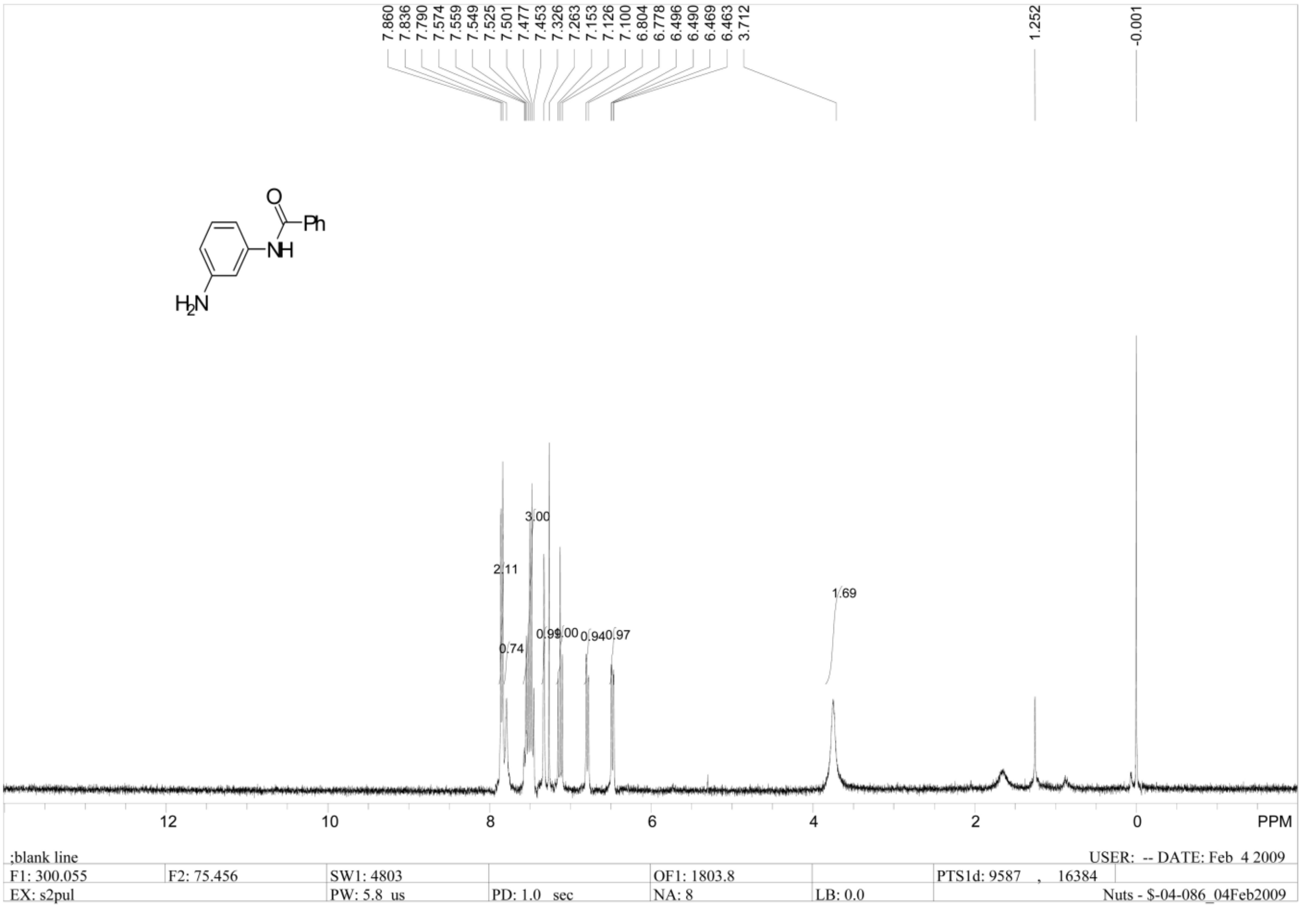
7.860
7.836
7.790
7.574
7.559
7.549
7.525
7.501
7.477
7.453
7.326
7.263
7.153
7.126
7.100
6.804
6.778
6.496
6.490
6.469
6.463
3.712
1.252
-0.001
O
Ph
NH
H_2N
3.00
2.11
1.69
0.74
0.99
1.00
0.94
0.97
12
10
8
6
4
2
0
PPM
;blank line
USER: -- DATE: Feb 4 2009
F1: 300.055
F2: 75.456
SW1: 4803
OF1: 1803.8
PTS1d: 9587 , 16384
EX: s2pul
PW: 5.8 us
PD: 1.0 sec
NA: 8
LB: 0.0
Nuts - $-04-086_04Feb2009

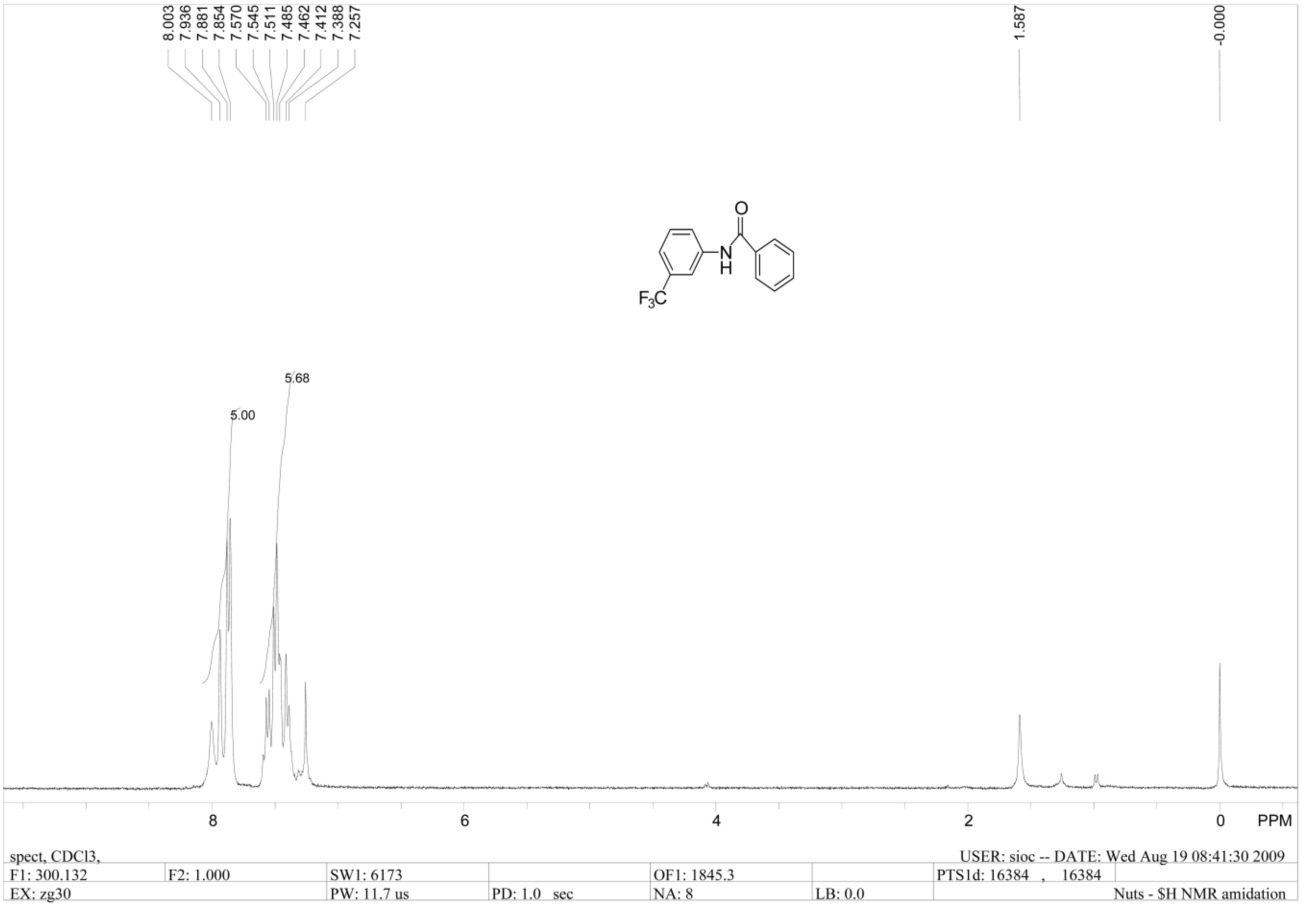
8.003
7.936
7.881
7.854
7.570
7.545
7.511
7.485
7.462
7.412
7.388
7.257
1.587
-0.000
O
N
H
F_3C
5.00
5.68
8
6
4
2
0
PPM
spect, CDCl3,
USER: sioc -- DATE: Wed Aug 19 08:41:30 2009
F1: 300.132
F2: 1.000
SW1: 6173
OF1: 1845.3
PTS1d: 16384 , 16384
EX: zg30
PW: 11.7 us
PD: 1.0 sec
NA: 8
LB: 0.0
Nuts - $H NMR amidation

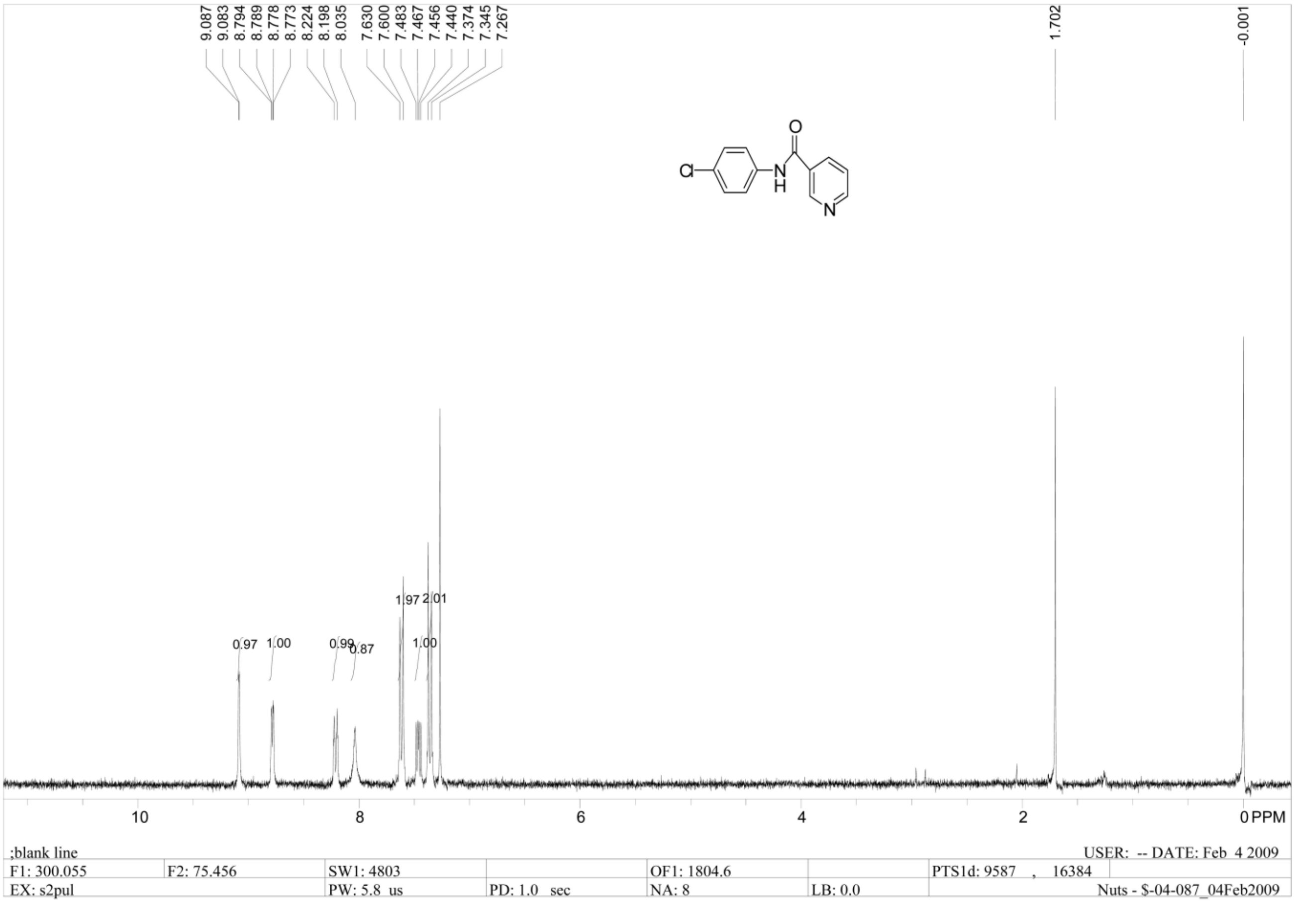
9.087
9.083
8.794
8.789
8.778
8.773
8.224
8.198
8.035
7.630
7.600
7.483
7.467
7.456
7.440
7.374
7.345
7.267
1.702
-0.001
O
Cl
N
H
N
0.97
1.00
0.99
0.87
1.97
2.01
1.00
10
8
6
4
2
0 PPM
;blank line
USER: -- DATE: Feb 4 2009
F1: 300.055
F2: 75.456
SW1: 4803
OF1: 1804.6
PTS1d: 9587 , 16384
EX: s2pul
PW: 5.8 us
PD: 1.0 sec
NA: 8
LB: 0.0
Nuts - $-04-087_04Feb2009

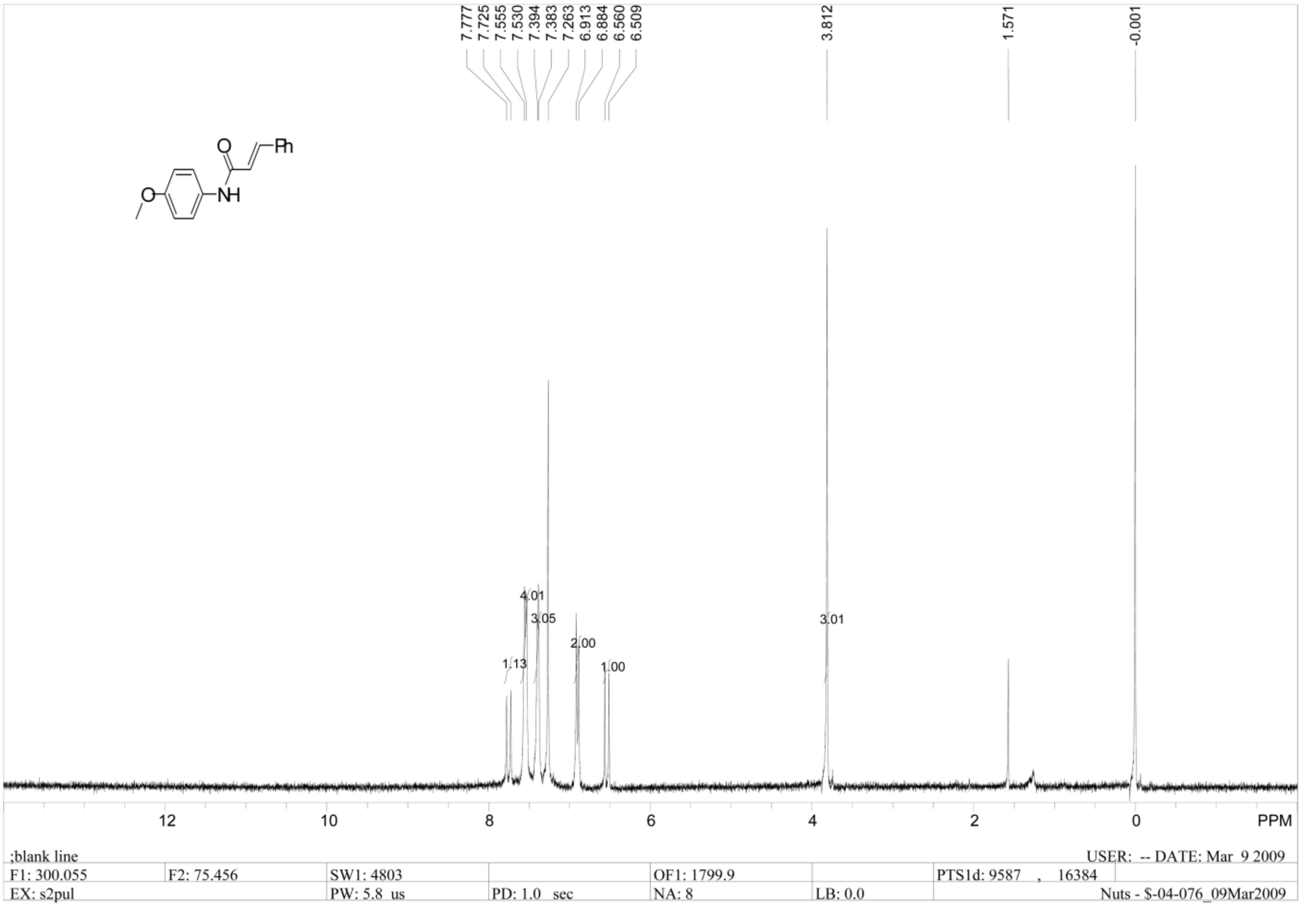

7.777
7.725
7.555
7.530
7.394
7.383
7.263
6.913
6.884
6.560
6.509
3.812
1.571
-0.001
O
Ph
O
NH
4.01
3.05
2.00
1.13
1.00
3.01
12
10
8
6
4
2
0
PPM
;blank line
USER: -- DATE: Mar 9 2009
F1: 300.055
F2: 75.456
SW1: 4803
OF1: 1799.9
PTS1d: 9587 , 16384
EX: s2pul
PW: 5.8 us
PD: 1.0 sec
NA: 8
LB: 0.0
Nuts - $-04-076_09Mar2009

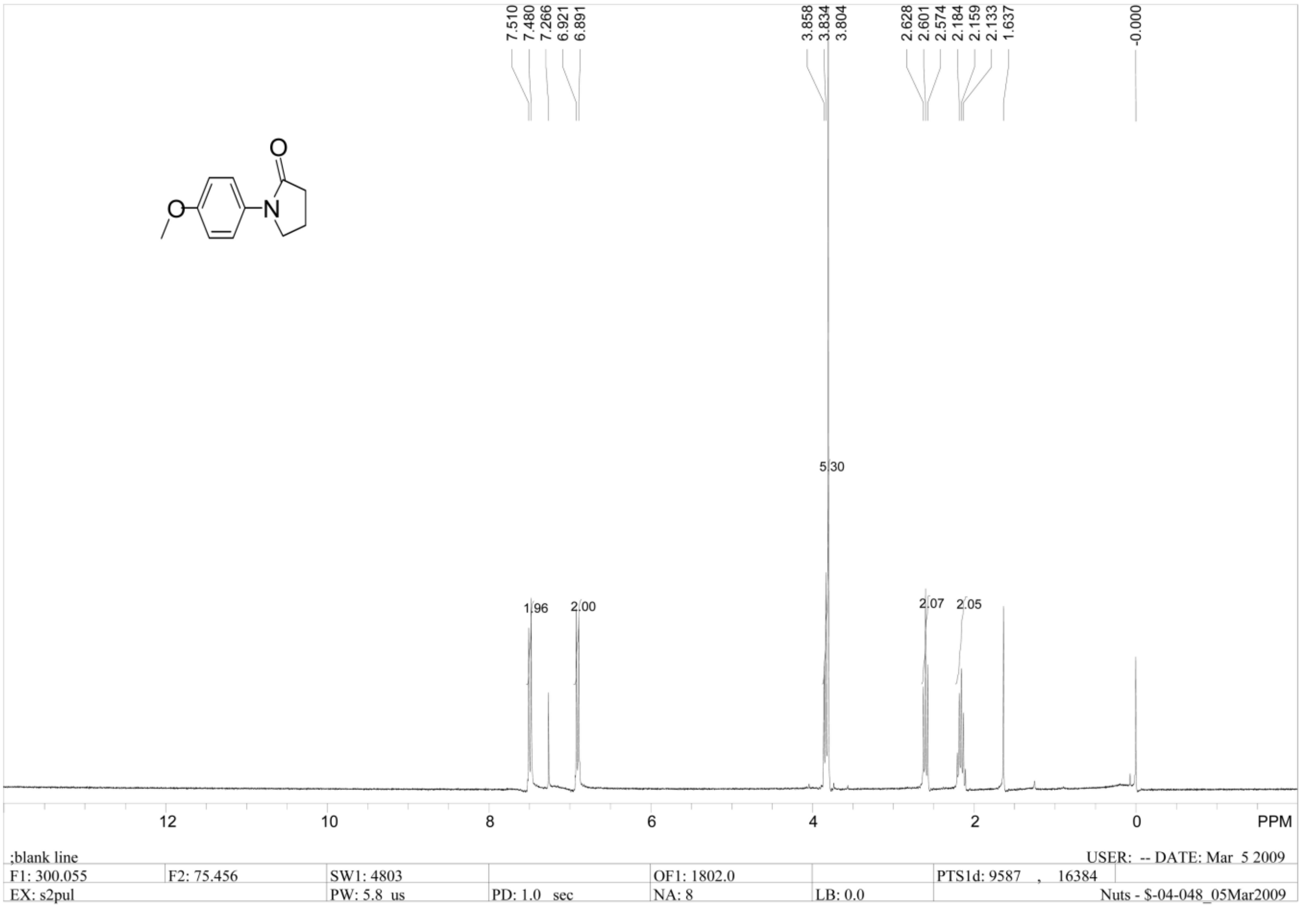

7.510
7.480
7.266
6.921
6.891
3.858
3.834
3.804
2.628
2.601
2.574
2.184
2.159
2.133
1.637
-0.000
O
O
N
5.30
1.96
2.00
2.07
2.05
12
10
8
6
4
2
0
PPM
;blank line
USER: -- DATE: Mar 5 2009
F1: 300.055
F2: 75.456
SW1: 4803
OF1: 1802.0
PTS1d: 9587 , 16384
EX: s2pul
PW: 5.8 us
PD: 1.0 sec
NA: 8
LB: 0.0
Nuts - $-04-048_05Mar2009

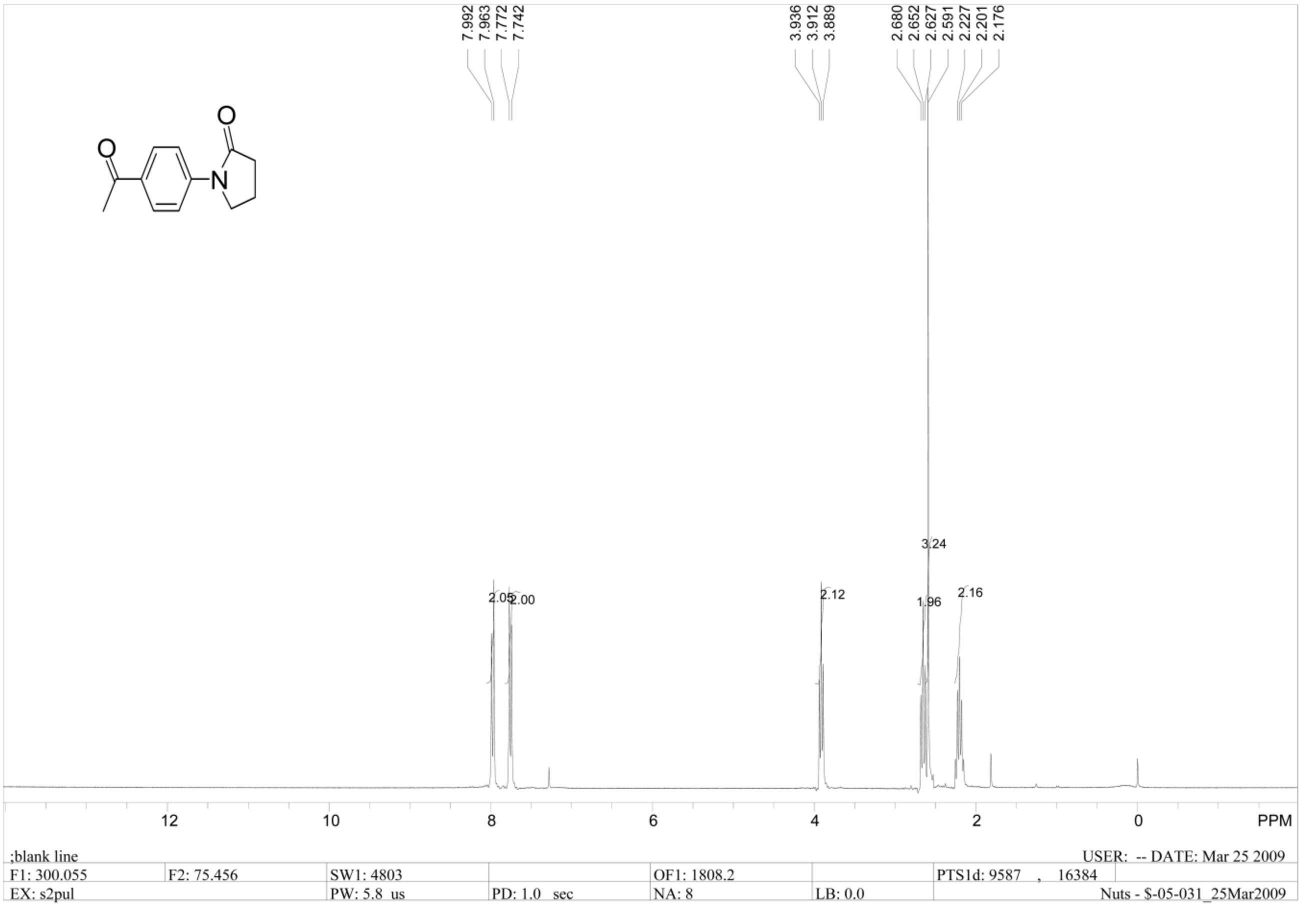
7.992
7.963
7.772
7.742
3.936
3.912
3.889
2.680
2.652
2.627
2.591
2.227
2.201
2.176
3.24
2.05
2.00
2.12
1.96
2.16
12
10
8
6
4
2
0
PPM
;blank line
USER: -- DATE: Mar 25 2009
F1: 300.055
F2: 75.456
SW1: 4803
OF1: 1808.2
PTS1d: 9587 , 16384
EX: s2pul
PW: 5.8 us
PD: 1.0 sec
NA: 8
LB: 0.0
Nuts - $-05-031_25Mar2009

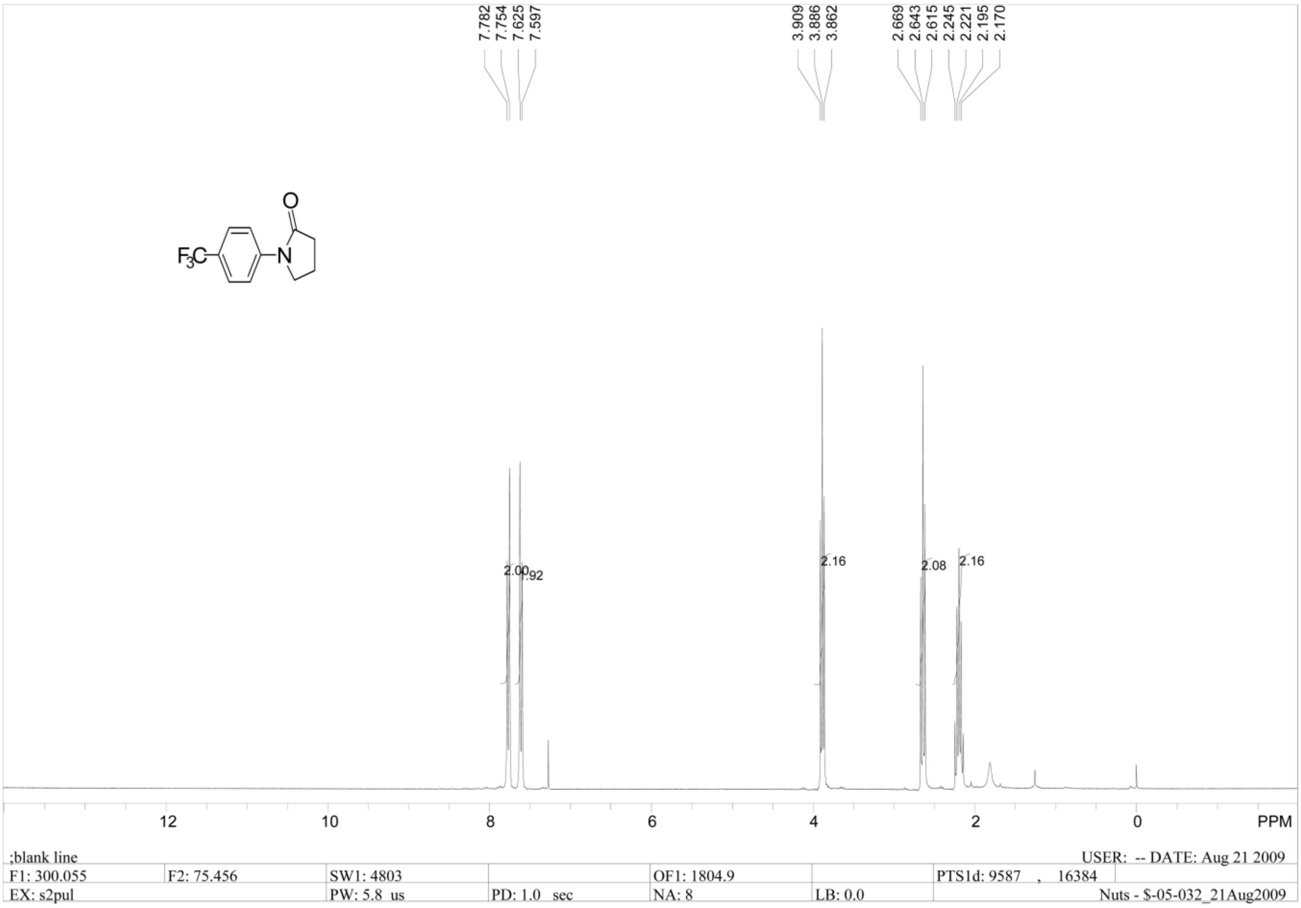
7.782
7.754
7.625
7.597
3.909
3.886
3.862
2.669
2.643
2.615
2.245
2.221
2.195
2.170
F3C
N
O
2.00
1.92
2.16
2.08
2.16
12
10
8
6
4
2
0
PPM
;blank line
USER: -- DATE: Aug 21 2009
F1: 300.055
F2: 75.456
SW1: 4803
OF1: 1804.9
PTS1d: 9587 , 16384
EX: s2pul
PW: 5.8 us
PD: 1.0 sec
NA: 8
LB: 0.0
Nuts - $-05-032_21Aug2009

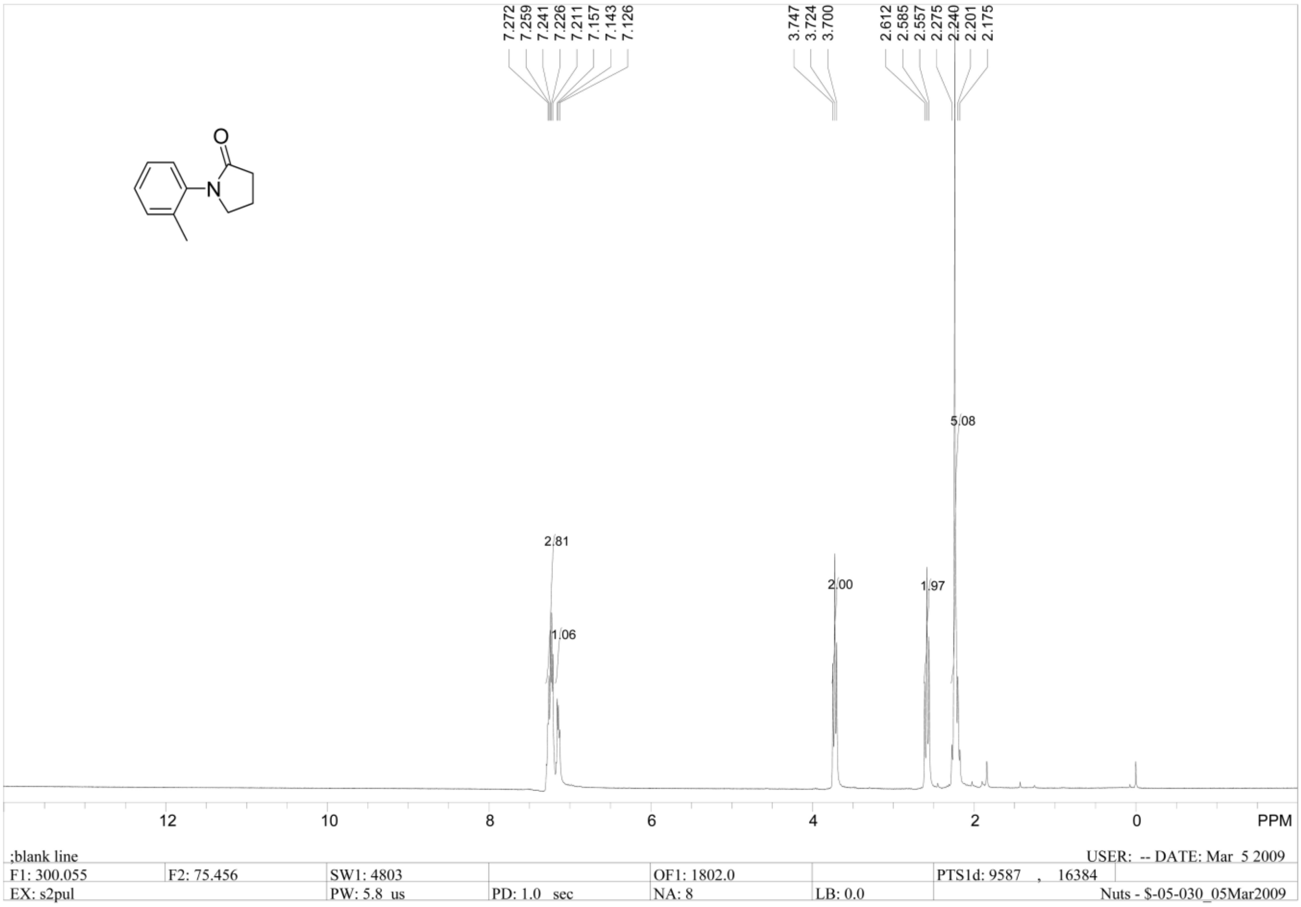
7.272
7.259
7.241
7.226
7.211
7.157
7.143
7.126
3.747
3.724
3.700
2.612
2.585
2.557
2.275
2.240
2.201
2.175
O
N
5.08
2.81
1.06
2.00
1.97
12
10
8
6
4
2
0
PPM
;blank line
USER: -- DATE: Mar 5 2009
F1: 300.055
F2: 75.456
SW1: 4803
OF1: 1802.0
PTS1d: 9587 , 16384
EX: s2pul
PW: 5.8 us
PD: 1.0 sec
NA: 8
LB: 0.0
Nuts - $-05-030_05Mar2009

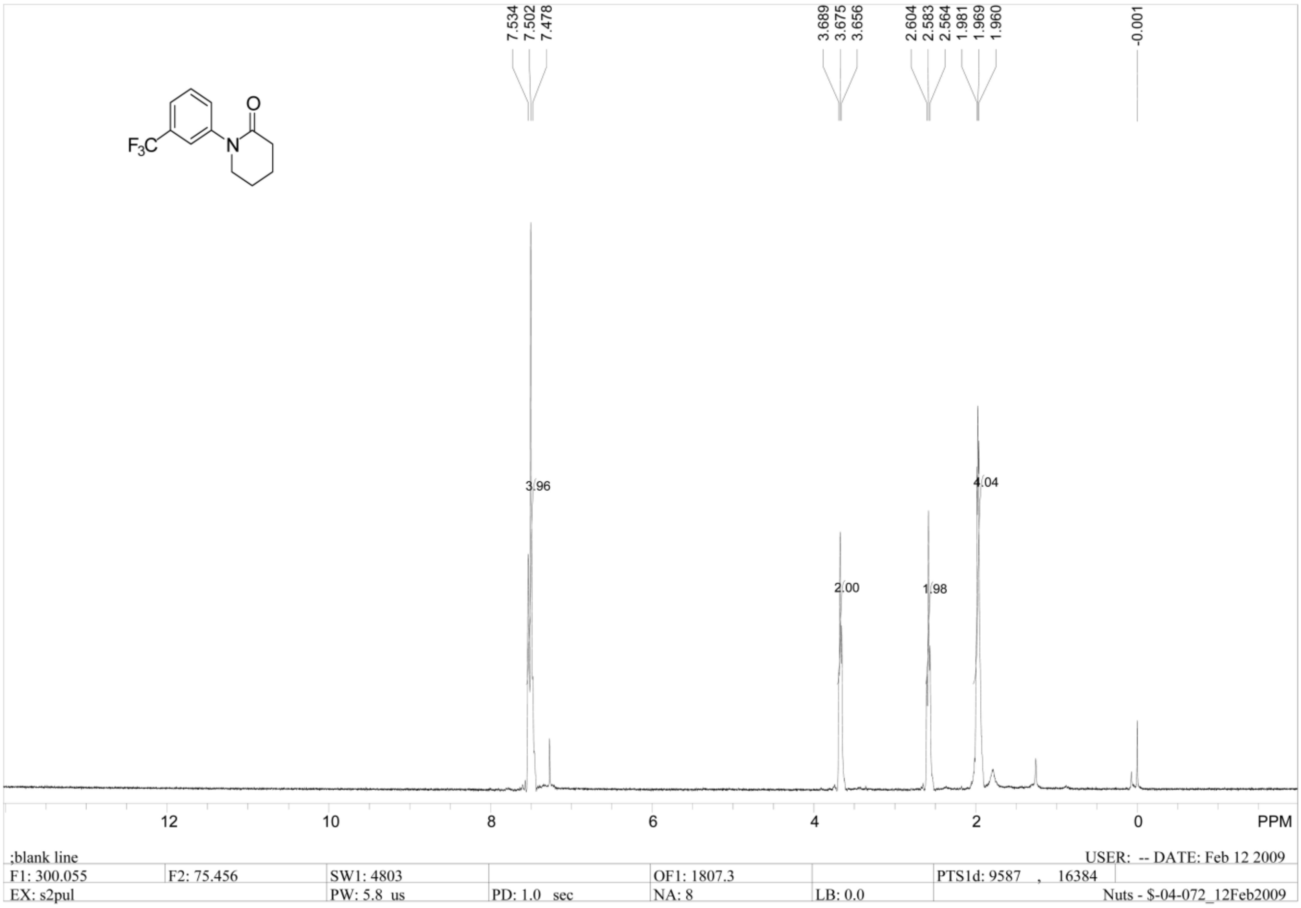
7.534
7.502
7.478
3.689
3.675
3.656
2.604
2.583
2.564
1.981
1.969
1.960
-0.001
F_3C
N
O
3.96
4.04
2.00
1.98
12
10
8
6
4
2
0
PPM
;blank line
USER: -- DATE: Feb 12 2009
F1: 300.055
F2: 75.456
SW1: 4803
OF1: 1807.3
PTS1d: 9587 , 16384
EX: s2pul
PW: 5.8 us
PD: 1.0 sec
NA: 8
LB: 0.0
Nuts - $-04-072_12Feb2009

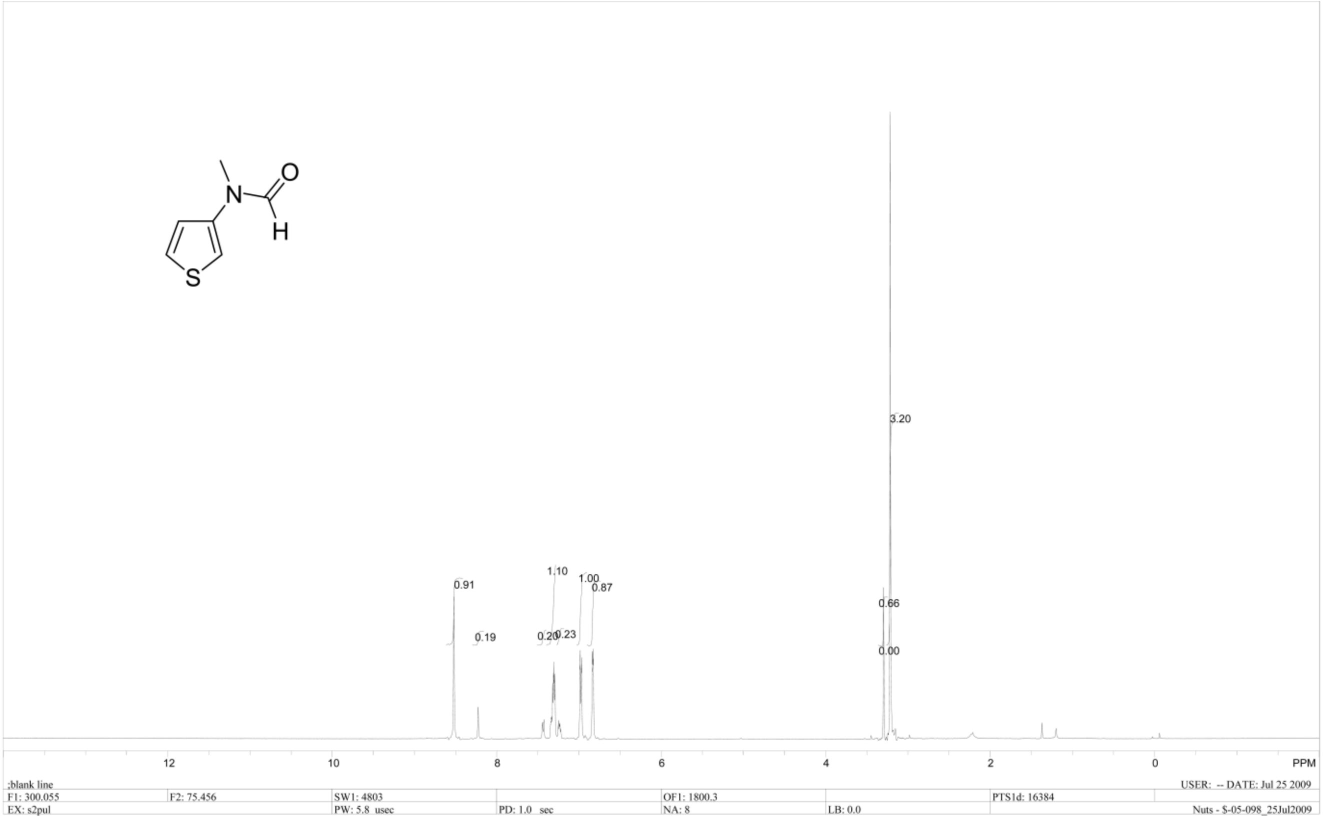
N
O
H
S
0.91
0.19
1.10
0.20
0.23
1.00
0.87
3.20
0.66
0.00
12
10
8
6
4
2
0
PPM
;blank line
USER: -- DATE: Jul 25 2009
F1: 300.055
F2: 75.456
SW1: 4803
OF1: 1800.3
PTS1d: 16384
EX: s2pul
PW: 5.8 usec
PD: 1.0 sec
NA: 8
LB: 0.0
Nuts - $-05-098_25Jul2009

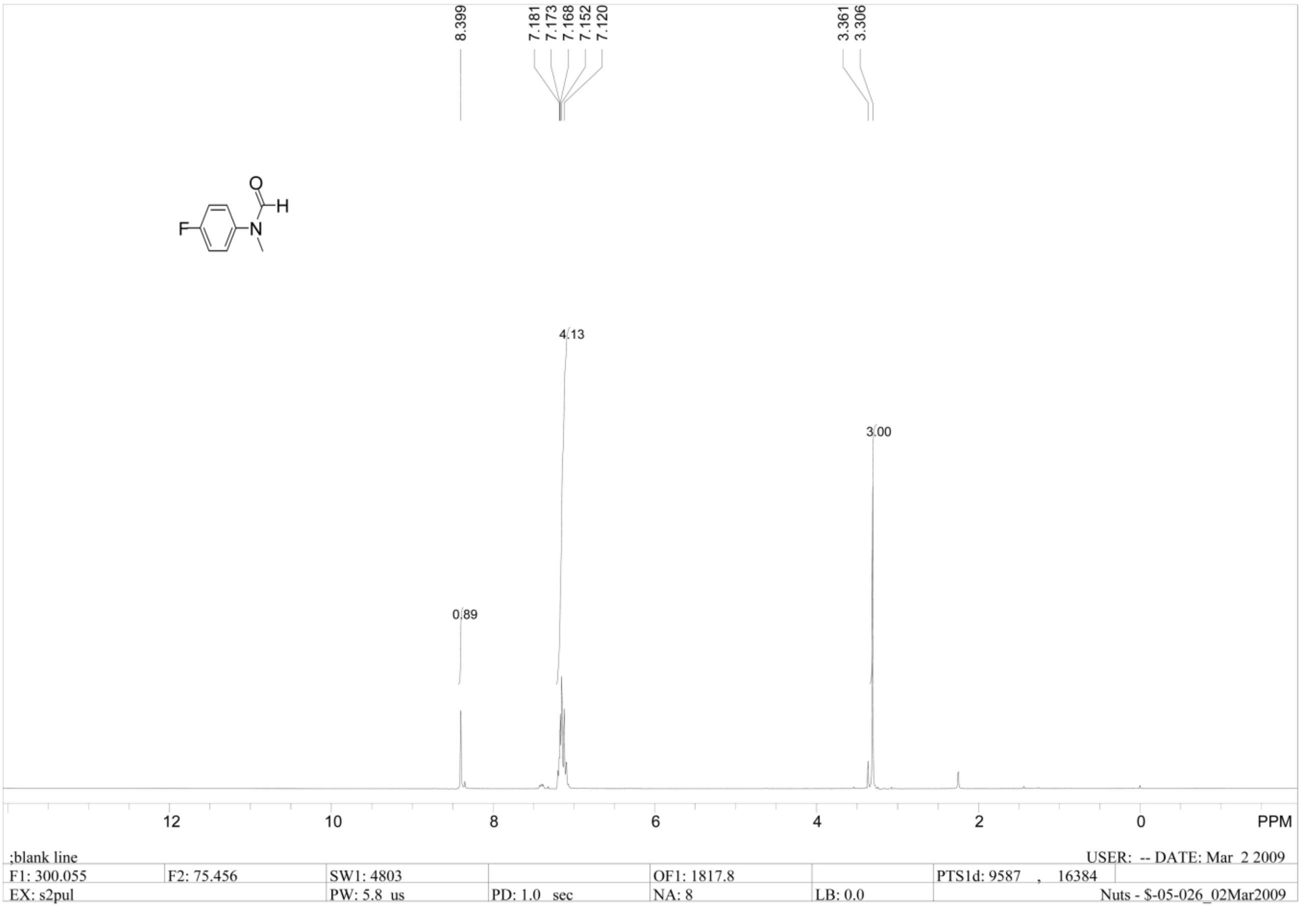
8.399
7.181
7.173
7.168
7.152
7.120
3.361
3.306
O
H
F
N
4.13
3.00
0.89
12
10
8
6
4
2
0
PPM
;blank line
USER: -- DATE: Mar 2 2009
F1: 300.055
F2: 75.456
SW1: 4803
OF1: 1817.8
PTS1d: 9587 , 16384
EX: s2pul
PW: 5.8 us
PD: 1.0 sec
NA: 8
LB: 0.0
Nuts - $-05-026_02Mar2009

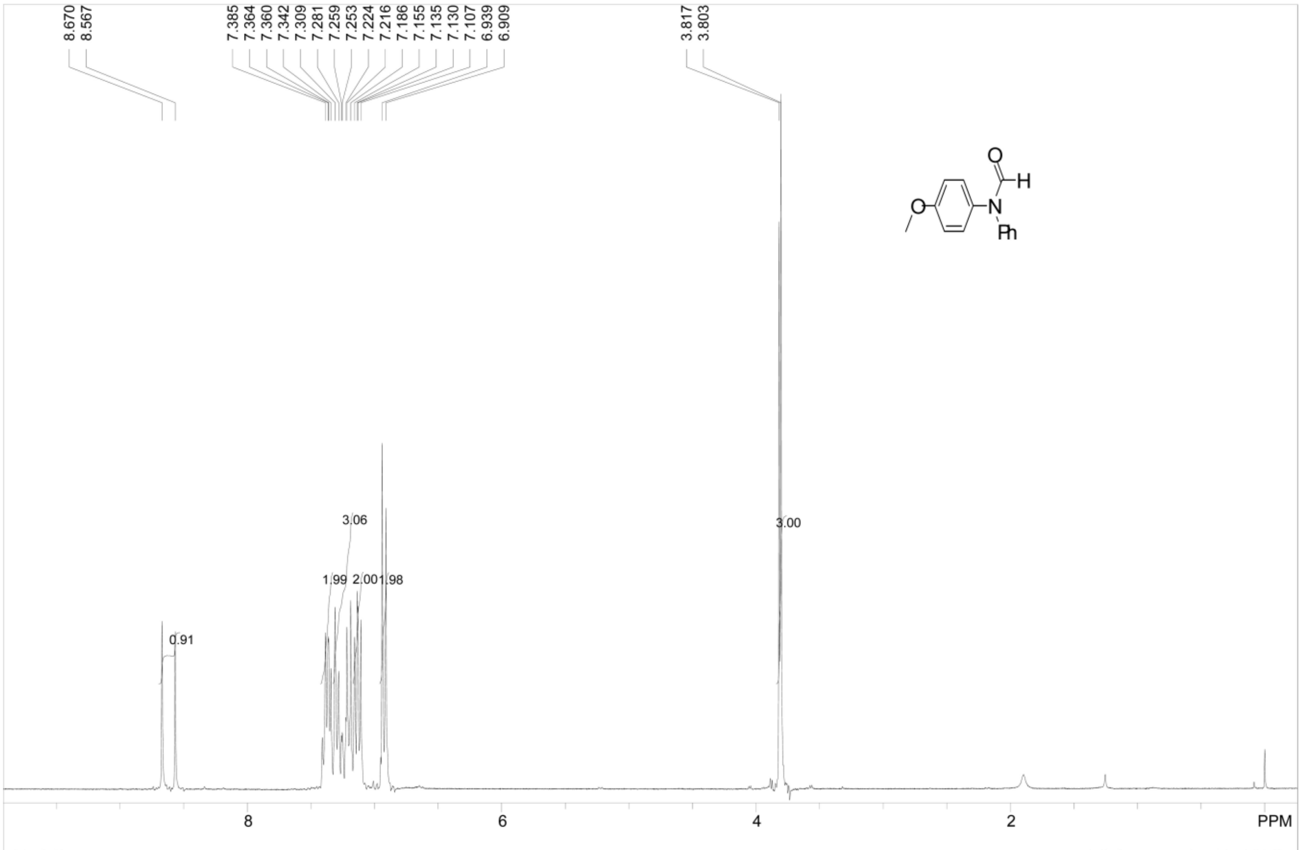

;blank line						USER: -- DATE: Feb 4 2009
F1: 300.055	F2: 75.456	SW1: 4803		OF1: 1803.2		PTS1d: 9587 , 16384
EX: s2pul		PW: 5.8 us	PD: 1.0 sec	NA: 8	LB: 0.0	Nuts - $-04-050_04Feb2009

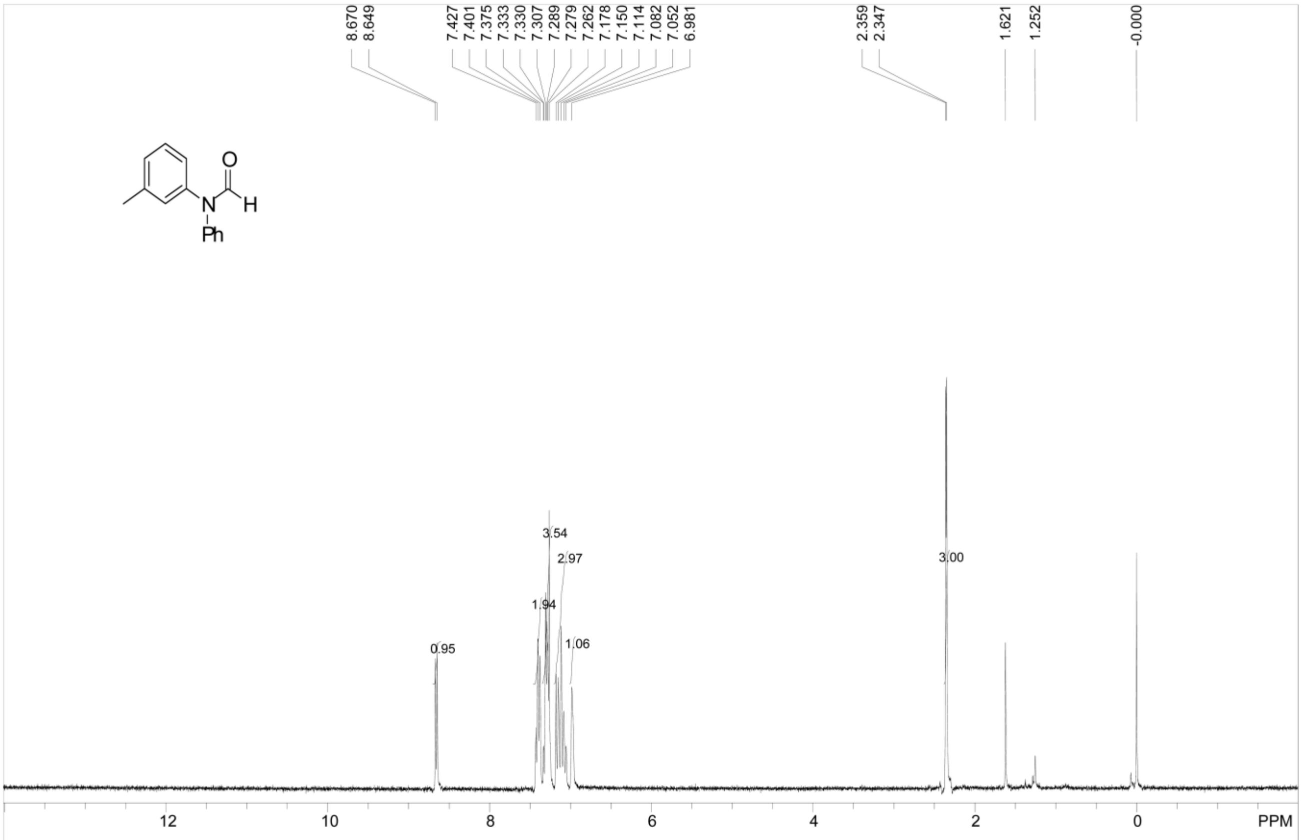

;blank line						USER: -- DATE: Feb 4 2009
F1: 300.055	F2: 75.456	SW1: 4803		OF1: 1802.9		PTS1d: 9587 , 16384
EX: s2pul		PW: 5.8 us	PD: 1.0 sec	NA: 8	LB: 0.0	Nuts - $-04-064_04Feb2009

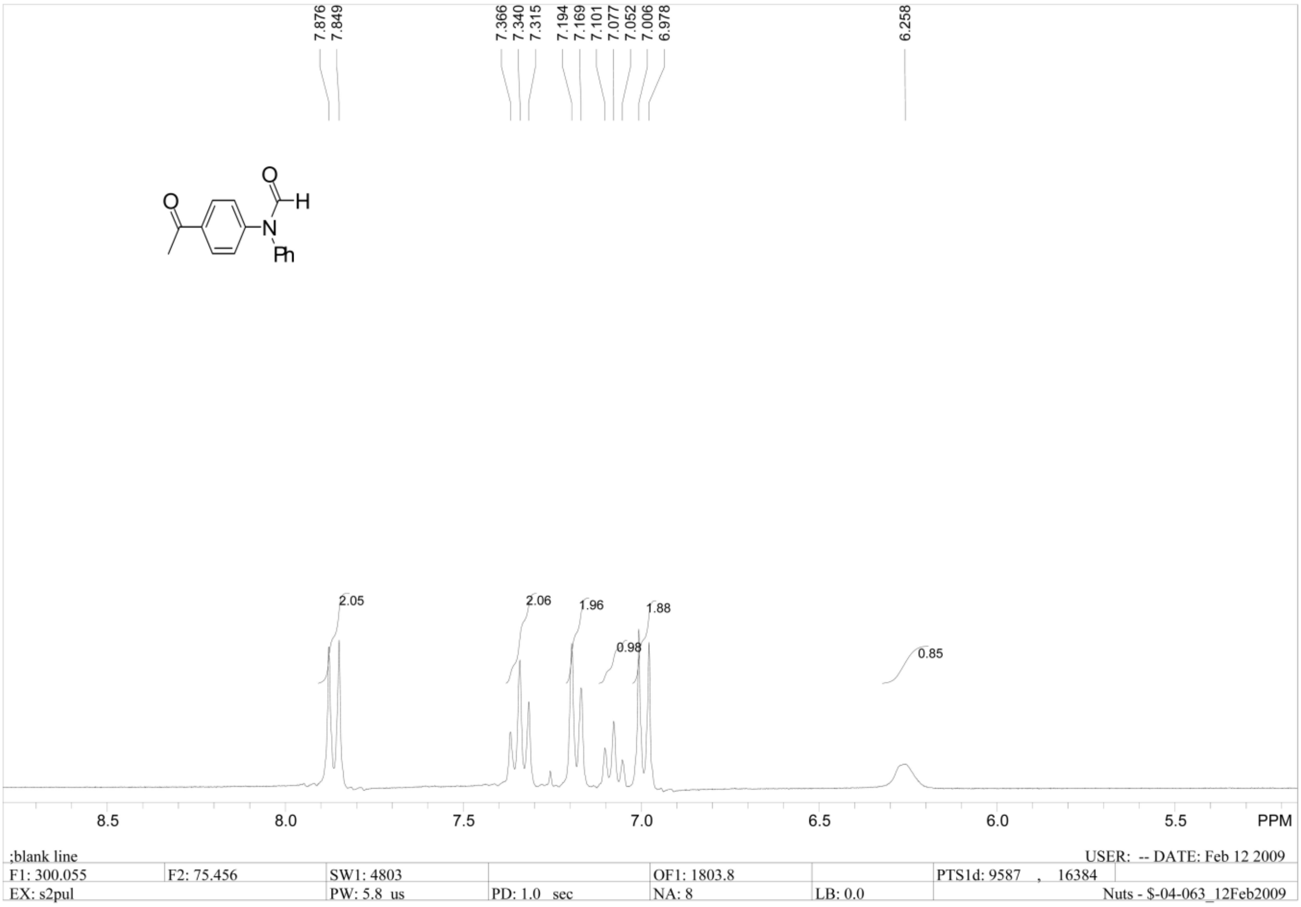
7.876
7.849
7.366
7.340
7.315
7.194
7.169
7.101
7.077
7.052
7.006
6.978
6.258
O
H
N
Ph
2.05
2.06
1.96
0.98
1.88
0.85
8.5
8.0
7.5
7.0
6.5
6.0
5.5
PPM
;blank line
USER: -- DATE: Feb 12 2009
F1: 300.055
F2: 75.456
SW1: 4803
OF1: 1803.8
PTS1d: 9587 , 16384
EX: s2pul
PW: 5.8 us
PD: 1.0 sec
NA: 8
LB: 0.0
Nuts - $-04-063_12Feb2009

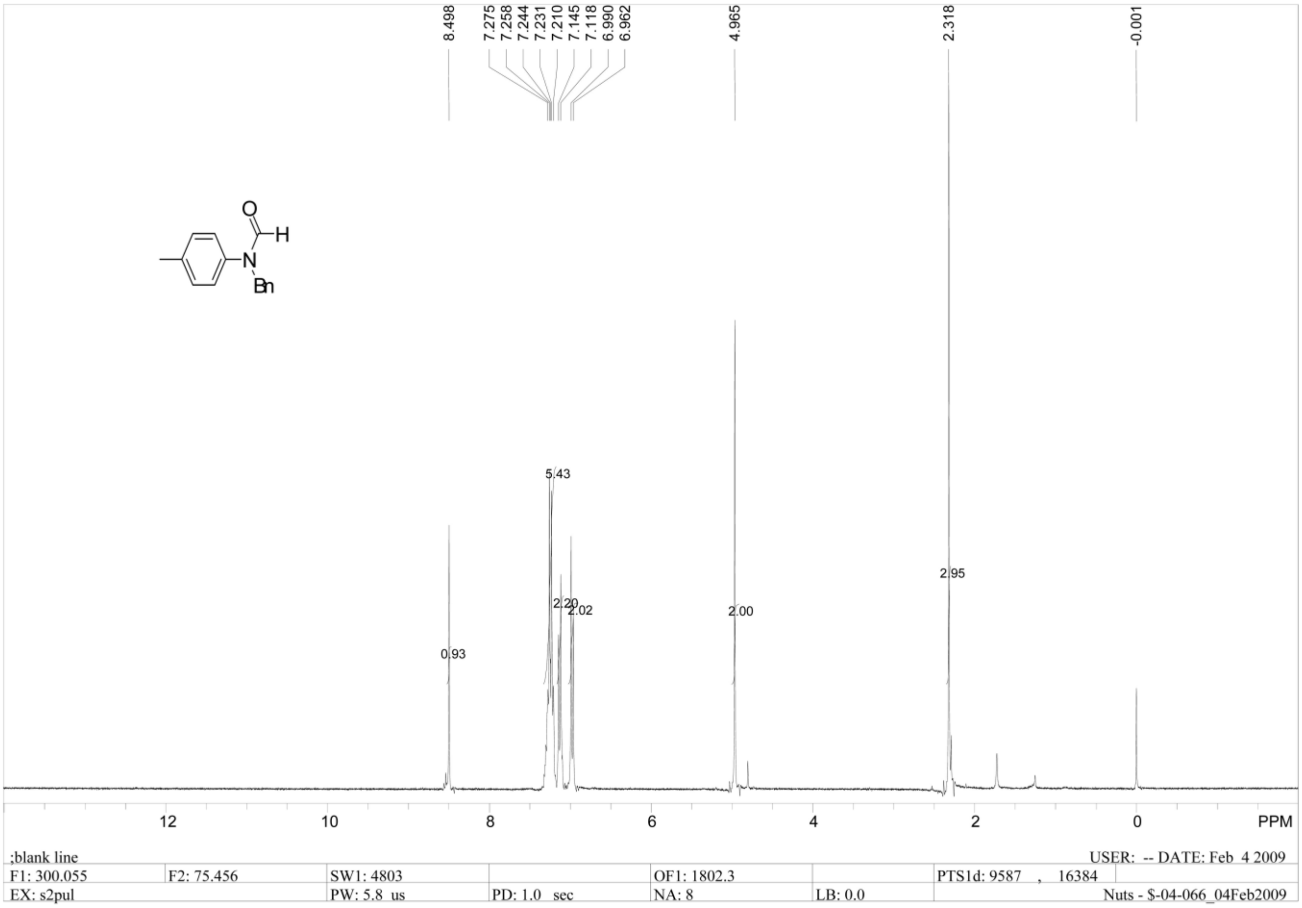
8.498
7.275
7.258
7.244
7.231
7.210
7.145
7.118
6.990
6.962
4.965
2.318
-0.001
O
H
N
Bn
5.43
2.20
2.02
0.93
2.00
2.95
12
10
8
6
4
2
0
PPM
;blank line
USER: -- DATE: Feb 4 2009
F1: 300.055
F2: 75.456
SW1: 4803
OF1: 1802.3
PTS1d: 9587 , 16384
EX: s2pul
PW: 5.8 us
PD: 1.0 sec
NA: 8
LB: 0.0
Nuts - $-04-066_04Feb2009

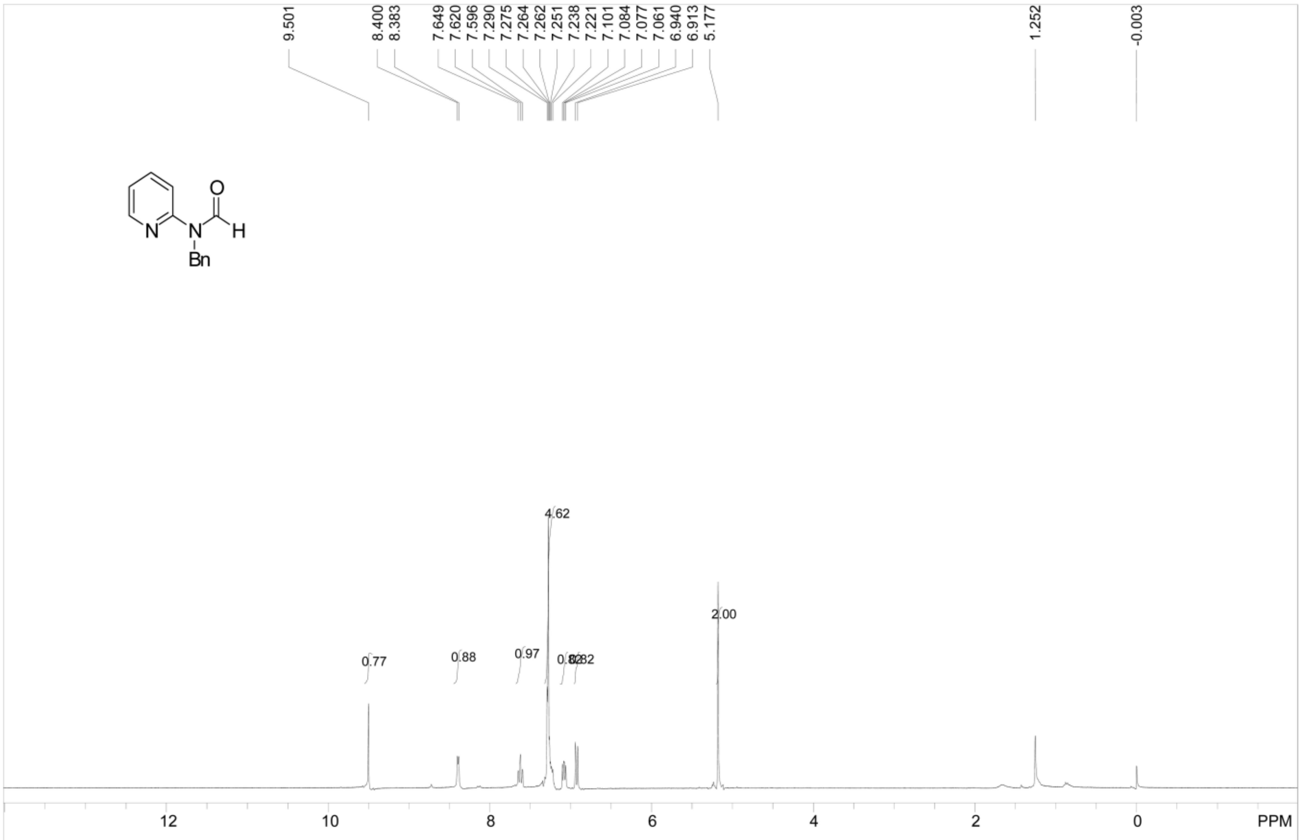

;blank line							USER: -- DATE: May 9 2009
F1: 300.055	F2: 75.456	SW1: 4803		OF1: 1804.6		PTS1d: 9587 , 16384	
EX: s2pul		PW: 5.8 us	PD: 1.0 sec	NA: 8	LB: 0.0		Nuts - $-05-004_09May2009

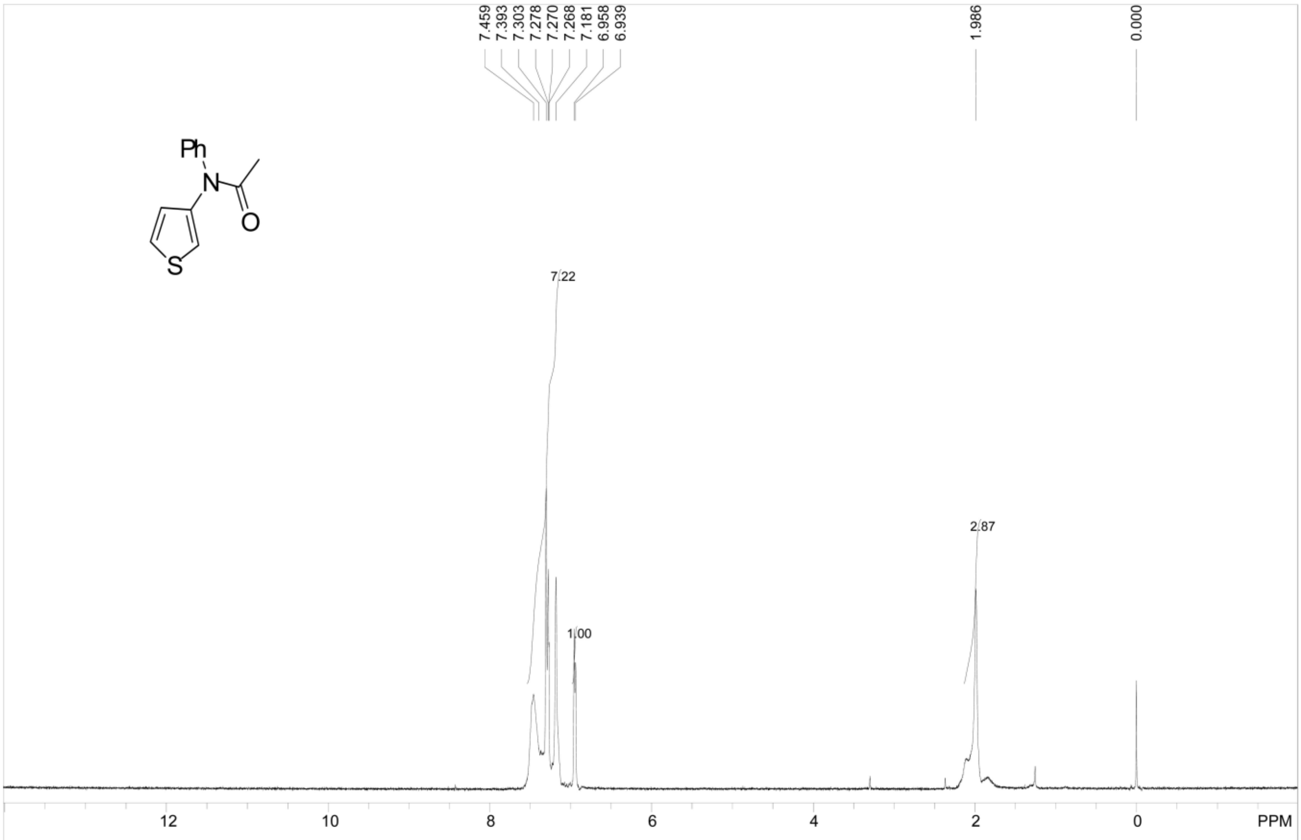

;blank line							USER: -- DATE: Feb 4 2009
F1: 300.055	F2: 75.456	SW1: 4803		OF1: 1804.6		PTS1d: 9587 , 16384	
EX: s2pul		PW: 5.8 us	PD: 1.0 sec	NA: 8	LB: 0.0		Nuts - $-04-051_04Feb2009

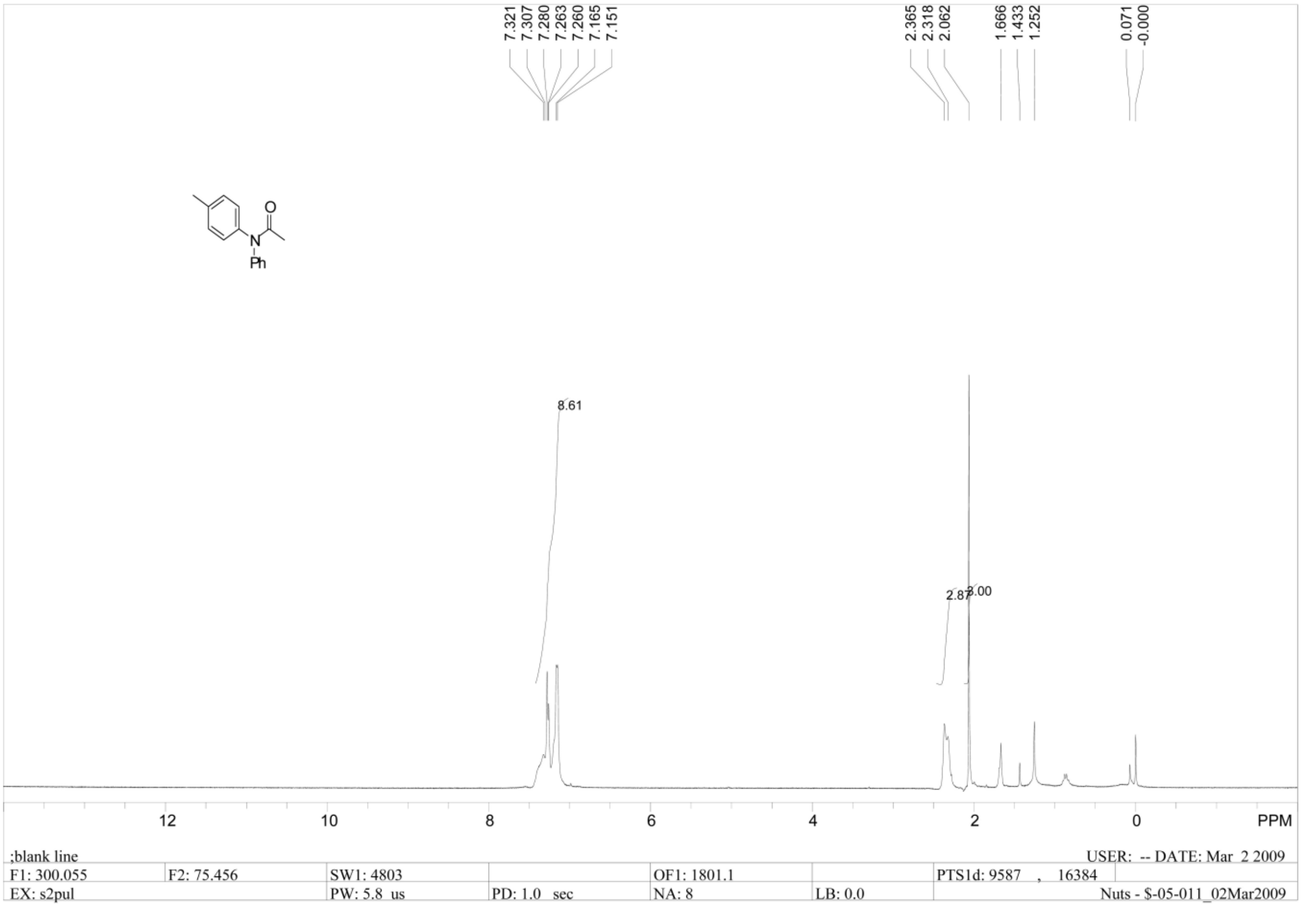
7.321
7.307
7.280
7.263
7.260
7.165
7.151
2.365
2.318
2.062
1.666
1.433
1.252
0.071
-0.000
O
N
Ph
8.61
2.87
3.00
12
10
8
6
4
2
0
PPM
;blank line
USER: -- DATE: Mar 2 2009
F1: 300.055
F2: 75.456
SW1: 4803
OF1: 1801.1
PTS1d: 9587 , 16384
EX: s2pul
PW: 5.8 us
PD: 1.0 sec
NA: 8
LB: 0.0
Nuts - $-05-011_02Mar2009

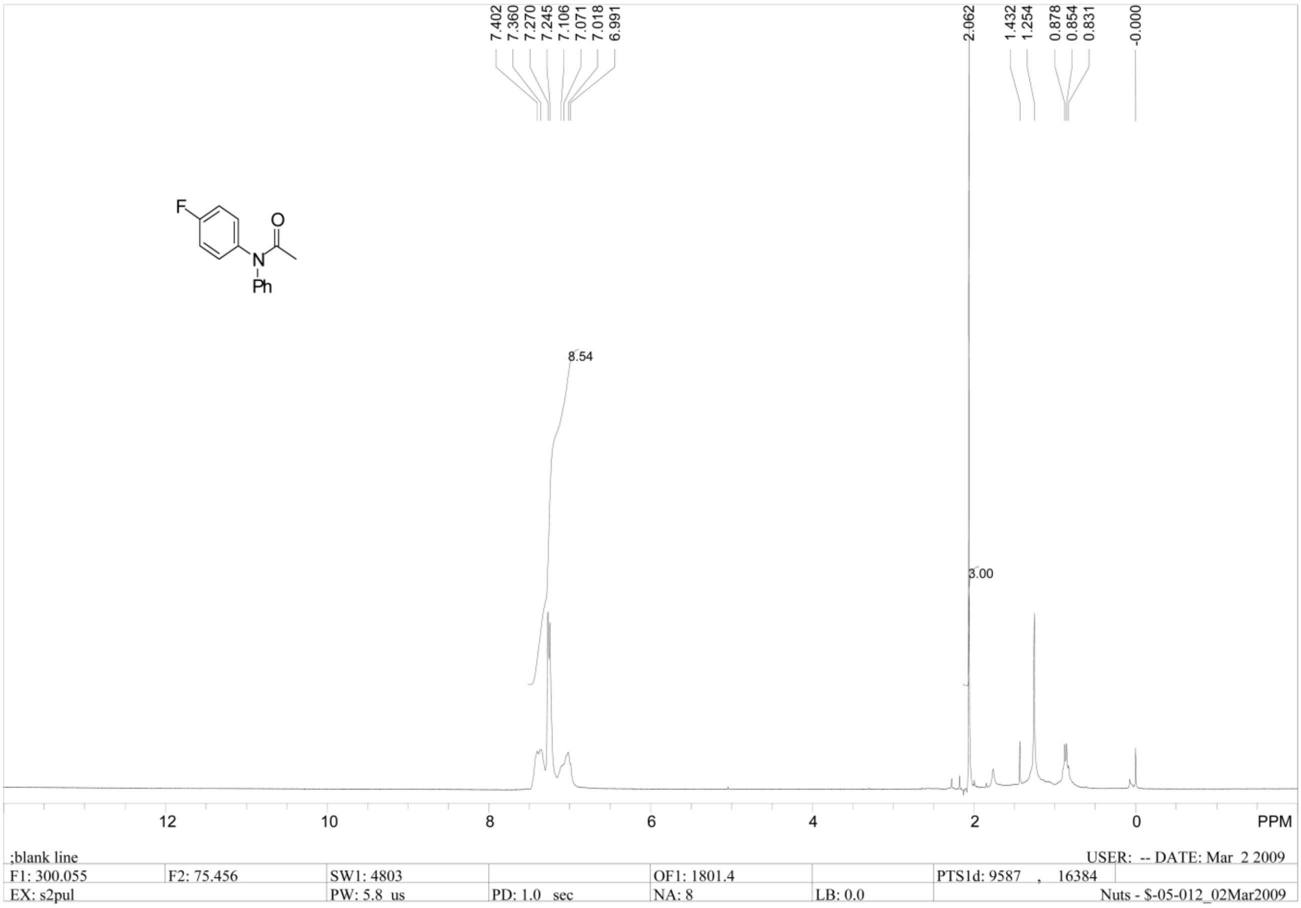
7.402
7.360
7.270
7.245
7.106
7.071
7.018
6.991
2.062
1.432
1.254
0.878
0.854
0.831
-0.000
F
O
N
Ph
8.54
3.00
12
10
8
6
4
2
0
PPM
;blank line
USER: -- DATE: Mar 2 2009
F1: 300.055
F2: 75.456
SW1: 4803
OF1: 1801.4
PTS1d: 9587 , 16384
EX: s2pul
PW: 5.8 us
PD: 1.0 sec
NA: 8
LB: 0.0
Nuts - $-05-012_02Mar2009

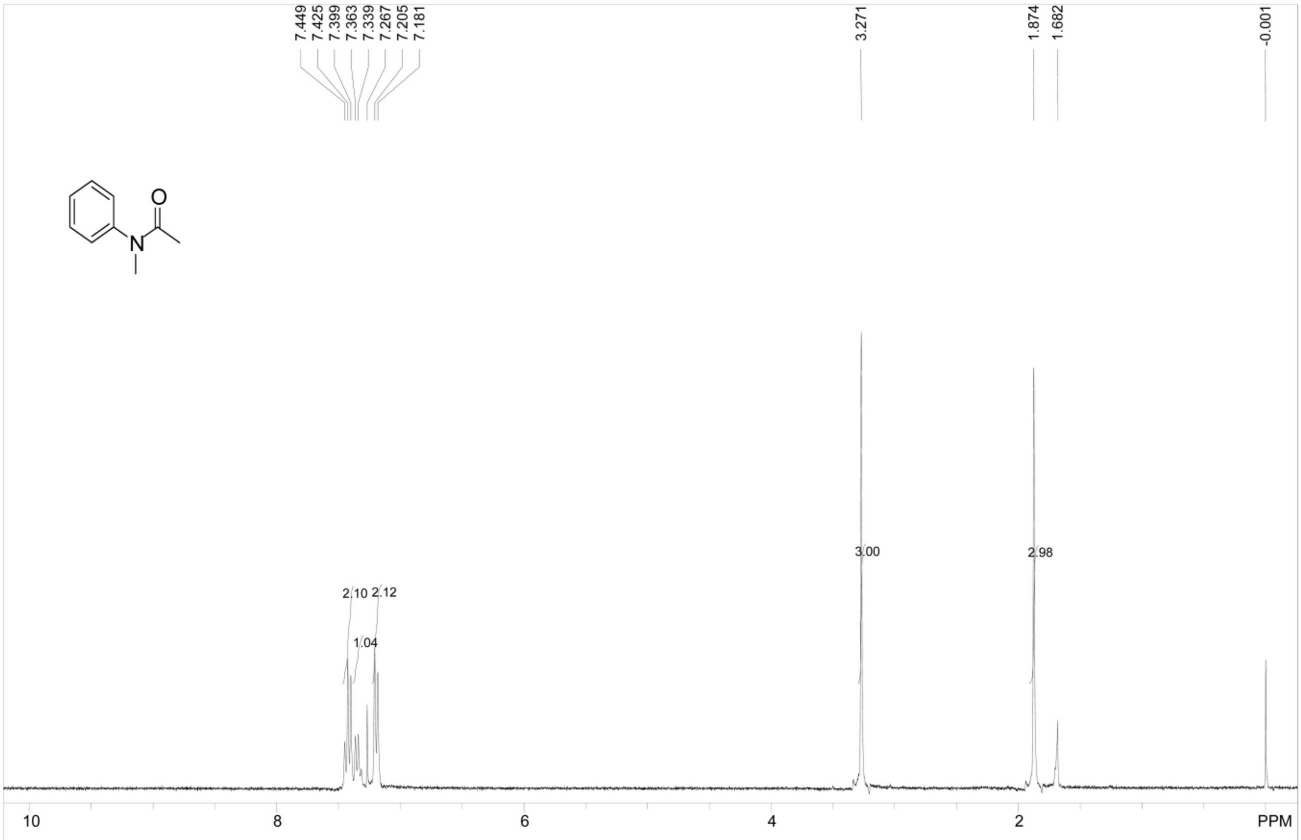

;blank line USER: -- DATE: Feb 12 2009

F1: 300.055	F2: 75.456	SW1: 4803		OF1: 1807.0		PTS1d: 9587 , 16384
EX: s2pul		PW: 5.8 us	PD: 1.0 sec	NA: 8	LB: 0.0	Nuts - $-05-005_12Feb2009

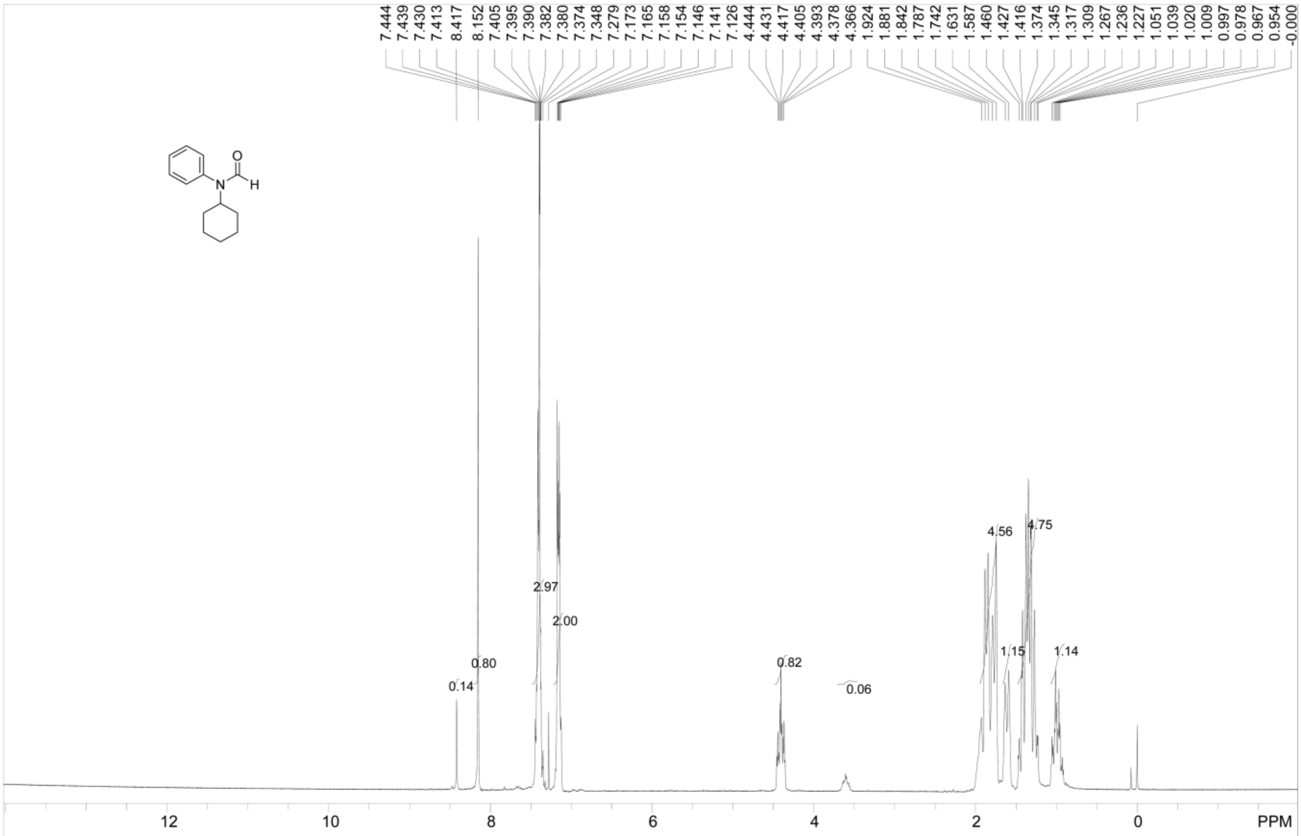

;blank line USER: -- DATE: Aug 21 2009

F1: 300.055	F2: 75.456	SW1: 4803		OF1: 1806.7		PTS1d: 9587 , 16384
EX: s2pul		PW: 5.8 us	PD: 1.0 sec	NA: 8	LB: 0.0	Nuts - $-05-050-1_21Aug2009

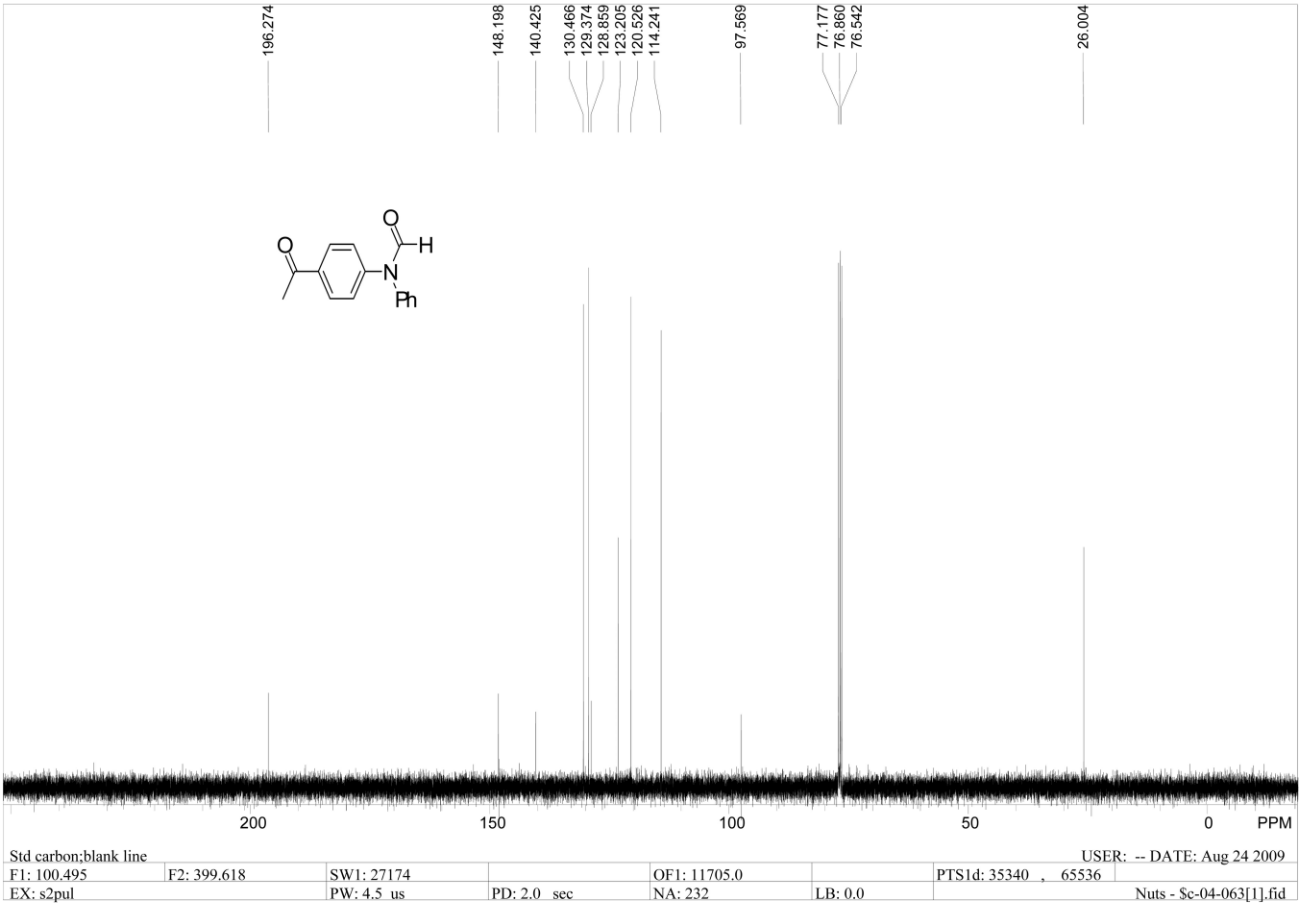
196.274
148.198
140.425
130.466
129.374
128.859
123.205
120.526
114.241
97.569
77.177
76.860
76.542
26.004
O
H
N
Ph
200
150
100
50
0
PPM
Std carbon;blank line
USER: -- DATE: Aug 24 2009
F1: 100.495
F2: 399.618
SW1: 27174
OF1: 11705.0
PTS1d: 35340 , 65536
EX: s2pul
PW: 4.5 us
PD: 2.0 sec
NA: 232
LB: 0.0
Nuts - $c-04-063[1].fid
